# Intestinal colonization of germ-free mice with indole-producing *E. coli* modulates the central and peripheral endocannabinoidome

**DOI:** 10.1016/j.jlr.2026.101070

**Published:** 2026-05-26

**Authors:** Giada Giorgini, Hayatte-Dounia Mir, Camille Etienne, Élizabeth Dumais, Elise Maximin, Cristoforo Silvestri, Nicolas Flamand, Laurent Naudon, Sylvie Rabot, Vincenzo Di Marzo

**Affiliations:** 1 Centre de Recherche de l’Institut Universitaire De Cardiologie Et De Pneumologie de Québec, Département of Médecine, Université Laval, Québec City, QC, Canada; 2 Canada Excellence Research Chair on the Microbiome-Endocannabinoidome Axis in Metabolic Health (CERC-MEND), Université Laval, Québec City, QC, Canada; 3 Joint International Unit between the CNR of Italy and Université Laval on Chemical and Biomolecular Research on the Microbiome and Its Impact on Metabolic Health and Nutrition (JIRU-MicroMeNu), Québec City, QC, Canada; 4 Université Paris-Saclay, INRAe, AgroParisTech, Micalis Institute, Jouy-en-Josas, France; 5 Institut sur la Nutrition et les Aliments Fonctionnels, Centre NUTRISS, École de Nutrition, Université Laval, Québec City, QC, Canada; 6 Université Paris-Saclay, INRAe, AgroParisTech, CNRS, Micalis Institute, Jouy-en-Josas, France

**Keywords:** Indole, Endocannabinoidome (eCBome), Germ-free mice, 2-Monoacylglycerols (2-MAGs), *N*-acylethanolamines (NAEs)

## Abstract

Colonization of germ-free (GF) mice with indole-producing *E*. *coli* exacerbates anxiety- and depression-like behaviors. Lack and subsequent reintroduction of gut microbiota in mice alter in opposing ways the brain and intestinal levels of the lipid mediators of the expanded endocannabinoid system, or endocannabinoidome (eCBome), which controls affective behaviors. By using LC/MS-MS-based targeted lipidomics and qPCR, we investigated the effect of GF mouse colonization with *E. coli* strains capable (wild-type, I+) or not (knock-out, I−) of producing indole, on the brain and intestinal eCBome. Compared to untreated GF mice, and unlike the I− strain, the I+ strain reduced the levels of 2-monoacylglycerols (2-MAGs), and 2-arachidonoylglycerol in particular, in the amygdala, and of 2-MAGs and NAEs in the hippocampus. Moreover, it elevated 2-MAGs in the ileum and reduced NAEs in the cecum. The mRNA expression of some eCBome receptors was also selectively altered by the I+ strain, including *Gpr55* (upregulated in the amygdala), *Gpr18* (downregulated in the ileum), *Cnr2* (upregulated in the colon), and *Gpr119* (downregulated in the cecum). LC/MS-MS-based targeted metabolomics detected the indole metabolite indoxyl-3-sulfate in the plasma and liver, but not brain, of I+-colonized mice. Our data pinpoint the eCBome as a potential mediator of the effects of indole.

Recent evidence suggests that signaling pathways controlling mammalian brain function, and in particular affective and cognitive behaviors, may be under the modulation of metabolites produced by bacteria stably living in the gut, also known as the gut microbiota (1). Typical metabolites, such as short-chain fatty acids (SCFAs), which are produced by several commensal bacteria as well as by probiotics like *Bifidobacteria* and *Lactobacilli*, affect anxiety- and depression-like behaviors in mice (2, 3, 4) and were suggested to exert beneficial effects in humans affected by mood disorders (5). Further, some GF mouse strains were previously shown to exhibit altered such behaviors (6) in a manner reversed by fecal matter transplant (FMT) (7, 8), thus establishing a clear link between the presence of commensal microorganisms and mood disorders as modeled in rodents. Recently, we have presented evidence that the tryptophan metabolite, indole, also produced by commensal bacteria such as *Escherichia coli*, (9) exacerbates anxiety-like and stress-induced depression-like behaviors when these bacteria are transferred into the gut of germ-free (GF) mice or when indole is introduced into the gut of conventionally raised specific pathogen-free mice or rats (9, 10, 11).

Among the signaling pathways strongly affected by gut microbiota and their metabolites, as well as by probiotics, the expanded endocannabinoid (eCB) system, or endocannabinoidome (eCBome), is one of the most recently discovered (12, 13, 14). This signaling system includes, among others, the two endogenous ligands of cannabinoid type-1 (CB1) and type-2 (CB2) receptors [or endocannabinoids (eCBs)], *N*-arachidonoyl-ethanolamine (anandamide, AEA) and 2-arachidonoyl-glycerol (2-AG), as well as their long chain fatty acid-derived congeners, the *N*-acyl-ethanolamines (NAEs) and 2-mono-acyl-glycerols (2-MAGs), respectively; the anabolic and catabolic enzymes common to each eCB and its congeners; and the receptors of all these mediators, which extend beyond CB1 and CB2. Indeed, the finding that: *1*) eCBs, like their congeners and other eCB-like mediators, may also bind to some non-cannabinoid receptors, such as other G-protein-coupled receptors (GPCRs), transient receptor (TRP) channels, and peroxisome proliferator-activated nuclear receptors (PPARs), and *2*) redundant anabolic and catabolic pathways and enzymes exist for NAEs and 2-MAGs, including AEA and 2-AG, justified the use of the word eCBome to encompass the whole signaling system including these molecules and their molecular targets and metabolic enzymes (15).

We have recently highlighted the existence of a close functional relationship between the eCBome and the gut microbiome (14). In particular, we and others have found that eCBome mediators such as 2-MAGs and NAEs can affect the taxonomic composition of the mouse gut microbiome (*i*) when administered to fecal microorganisms in vitro (16, 17) and (*ii*) in vivo, in mice with genetic deletion of eCBome metabolic enzymes (16, 18, 19, 20). On the other hand, the gut microbiome also directly affects the eCBome, as shown by the finding that: *1*) GF mice exhibit altered intestinal and brain levels of eCBome receptor and/or mediators in manner reversed one week after FMT in male mice (21, 22); *2*) typical gut bacterial metabolites such as SCFAs (23, 24) and lipopolysaccharides (LPS) (25, 26, 27) alter eCB and eCB-like mediator receptor and metabolic enzyme expression in cell cultures or in tissues in vivo; and *3*) commensal bacteria produce eCB-like molecules capable of acting at the host eCBome receptors (28, 29, 30, 31). Indeed, some actions of gut microbiota are accompanied by changes in cellular, tissue, and organismal functions that can be reconducted with modulation of eCBome signaling, including metabolic (32, 33) and behavioral (34, 35) effects.

In the present study, we aimed at investigating the possibility that indole, produced by various commensal bacterial species inhabiting the mouse gut, alters the brain and intestinal eCBome. We have used *E. coli* as a typical model species for indole production (9) and have colonized GF mice with either a naturally indole-producing (I+) strain (wild-type *E. coli*), or a knock-out strain (I−), which cannot produce this tryptophan metabolite. We have then analysed by LC/MS-MS the concentrations of NAEs and 2-MAGs in the plasma and several brain areas and intestinal sections of these two groups of mice, as well as the mRNA expression of the major eCBome receptors and metabolic enzymes in these tissues, as compared to untreated GF mice of the same age, sex and strain.

## Materials and Methods

### Animals

Twenty 4-week-old male GF mice of the C57BL/6J strain were obtained from the GF rodent breeding facility Anaxem (Germfree animal facilities of the Micalis Institute, INRAe, France). They were randomly separated in three groups of 6–8 mice and transferred in three sterile isolators (Getinge), fitted with DPTE® aseptic transfer systems (Getinge) allowing sterile connection of containers (Getinge) to import sterile consumables. The isolators were ventilated with HEPA-filtered sterile air under positive pressure. Inside the isolators, the animals were housed in collective cages (3–4 mice/cage) containing sterile bedding made of wood shavings. They had free access to autoclaved tap water and a γ-irradiated (45 kGy) standard diet (R03; Scientific Animal Food and Engineering, Augy, France). The animal room was maintained at 20–24°C and kept on a 12 h light/dark cycle (lights on at 7:30 a.m.).

### Bacterial strains and animal experimental design

The National Institute of Genetics (Mishima, Japan) supplied the wild-type *E. coli* BW25113 strain and the JW3686 mutant invalidated for the *tnaA* gene, which codes for the enzyme responsible for indole production from tryptophan (36). Strains were stored at −80°C in Brain Heart Infusion broth supplemented with glycerol (final concentration 40%). For colonization of GF mice, they were grown in lysogenic broth overnight at 37 °C under aerobic conditions. The cultures were then divided into sterile vials and transferred under sterile conditions to the isolators (Supplemental Fig. S1).

In one isolator, mice remained GF, and the GF status was monitored every 2 weeks by microscopic examination and aerobic and anaerobic cultures of samples of freshly voided feces. In the two other isolators, mice colonization was achieved by spreading the *E. coli* cultures on the mice's fur and in the cage environment. The mice mono-associated with the wild-type strain or with the mutant strain were named “I+” mice and “I−” mice, respectively. The level of gut bacterial population was determined 2 weeks after colonization and one week before killing by cultures of serial dilutions of freshly voided feces. At the same time point, fresh feces were also collected from each mouse for tryptophan and indole analysis by HPLC with fluorescence detection (9).

At the age of 13 weeks, mice were weighed, anaesthetized with isoflurane and killed by decapitation. The truncal blood was collected in a tube coated with sodium EDTA 0.5 M; after centrifugation (3000g, 20 min, 4°C), the plasma was aliquoted into cryotubes and frozen at −80°C. Brains were quickly removed from the craniums, placed 1 min at −30°C in isopentane, then stored at −80°C. The gastro-intestinal tract was removed and dissected on ice. The cecum was opened, the cecal content was removed, weighed, aliquoted into cryotubes and frozen at −80°C. The small intestine and the colon were opened longitudinally, and the residual digestive content was removed gently. One-cm long tissue pieces were excised from the duodenum (3 consecutive pieces, starting excision 1-cm below the gastroduodenal junction), the jejunum (3 consecutive pieces, starting excision 2-cm after the end of the duodenum), the ileum (4 consecutive pieces, starting excision 1-cm above the ileocolic junction), the cecum (3 adjacent pieces), and the colon (3 consecutive pieces, starting excision 2-cm after the cecocolic junction). Among the tissue pieces collected from each intestinal section, one was transferred into cryotubes filled with RNAlater™ stabilization solution, while the others were transferred into empty vials. All those intestinal tissue samples were subsequently stored at −80°C until analyses. At the same time, a piece of colonic tissue that had been excised 1 cm from the cecocolic junction was transferred into DMEM (Dulbecco’s Modified Eagle Medium)-0% glucose for instant intestinal permeability measurement in Ussing chambers, as described elsewhere (37). The mice were not fasted prior to sacrifice. The sacrifices took place over two consecutive mornings, starting at 9:30 a.m. and concluding by noon. On each day, 10 mice were sacrificed, with a 15-min interval allotted for each mouse. Specifically, during these two days, 3 GF mice out of a total of 6, 3 I− mice out of 6, and 4 I+ mice out of 8 were sacrificed. The mice from each group were interspersed to minimize potential bias related to the timing of the sacrifices. The same procedure was applied to the cohort used for metabolomics analysis; on both days, 4 I− mice and 4 I+ mice were sacrificed, again interspersed to minimize bias related to the timing of the sacrifices.

All procedures were carried out in accordance with the European guidelines for the care and use of laboratory animals and approved by the ethics committee of the INRAe Research Center at Jouy-en-Josas (approval reference: APAFIS#9909–201704201500513 v2).

### Gene expression analysis by quantitative PCR

Brain samples were collected and processed as described previously (38, 39, 40, 41, 42, 43). Bilateral 14-gauge punches were collected from 1-mm coronal slices with a Stainless-steel brain matrice on dry ice and stored at −80°C for further analysis. RNA was isolated by TRIzol homogenization, quantified and retrotranscribed (High-Capacity cDNA Reverse Transcription Kit, Life Technologies) using the manufacturer protocols. Quantitative real-time PCR was carried out in CFX Opus 384 Real-Time PCR System (Biorad) by using SYBR Green, Abclonal detection. Selective primers were purchased from Integrated DNA Technologies (Supplemental Table S1). Data normalization was performed by using *Gadph*, relative quantification assessed by the 2^−ΔΔCt^ formula.

### Quantification of lipid mediators by HPLC-MS/MS analysis

For tissue samples, lipids extraction was performed as in PMID 31690638 (21) with slight modifications. Briefly, approximately 10 mg of each intestinal and brain tissue were homogenized using a tissue grinder, then suspended in 0.5 ml of Tris-HCl (50 mM pH 7.4). 0.5 ml methanol containing 5 ng of deuterated standards and acetic acid (0.5%) was next added to the mixture. An organic phase extraction with chloroform was performed on each sample by adding 1 ml of chloroform in the mixture, vortexing for 30 s and centrifuging at 4,000g for 5 min. This was repeated three times for a total of 3 ml of chloroform. The organic phases were then pooled and evaporated under reduced pressure using a SpeedVac evaporator. For plasma samples, lipids were extracted as in PMID 32012340 (44) with slight modifications. 40 μl of plasma samples were mixed with 460 μl of TRIS (pH 7.4, 50 mM). Toluene (2 ml) containing the ISTDs was then added to the samples, vortexed for 1 min, then centrifuged at 4,000 *g* for 5 min without brakes. Samples were then placed in an ethanol-dry-ice bath (−80°C) to freeze the aqueous phase (bottom). The organic phase (top) was then collected and evaporated to dryness under a stream of nitrogen. The lipid extracts were next dissolved in 60 μl of mobile phase containing 50% of solvent A (water + 1 mM ammonium acetate + 0.05% acetic acid) and 50% of solvent B (acetonitrile/water 95/5 + 1 mM ammonium acetate + 0.05% acetic acid). 40 μl of each sample was injected into an HPLC column (Kinetex C8, 150 × 2.1 mm, 2.6 μm, Phenomenex) and eluted at a flow rate of 400 μl/min using a discontinuous gradient of solvent A and solvent B.

Quantification of lipid mediators (Supplemental Table S2) was carried out by HPLC interfaced with a Shimadzu 8,050 triple quadrupole mass spectrometer and using multiple reaction monitoring for the compounds and their deuterated homologs or a surrogate. Only peaks having the same retention time as the purified compounds of interest having and a signal-to-noise ratio greater than 5 were kept for quantification. In the case of MAGs, the data are presented as 2-MAGs but represent the combined signals from the *sn*-2- and *sn*-1(3)-isomers because the latter are most likely generated from the former via acyl migration (Supplemental Table S2).

### Statistical analysis

Statistical analysis was performed using GraphPad Prism version 10.4.0. Data are expressed as the medians ± SD of n = 6–8 mice/samples per group. Outliers were identified by the ROUT test (Q = 10%). The standard score (Z-score) method was used to calculate an overall median for 2-MAGs and NAEs, considering the contribution of each congener identified by our approach in plasma, brain and the intestine. Data for single mediators were compared using one-way ANOVA followed by Tukey test or Kruskal-Wallis tests followed by Dunn's post-test for multiple comparisons. Because the data distribution and intra-group variability were borderline, either a parametric or a non-parametric type of statistical test could be used. However, based on the fact that our mice are inbred, the distribution of the lipids should have been normal. For this reason, both tests were used. Results were considered statistically significant if *P* < 0.05.

### Targeted metabolomics analysis of tryptophan metabolites

Plasma, liver and brain samples (n = 8 for each tissue and each group of mice) from a different cohort of GF mice colonized with either the I+ or I− *E. coli* strains were extracted, processed and analysed for the presence of tryptophan and metabolites by LC/MS-MS at the Plateforme de Métabolomique et d’Analyses Chimiques (PMAC), *Faculté de Médecine de Tours*, *France*. For plasma extraction, the samples were vortexed vigorously and 200 μl of internal solution in MeOH were added to 50 μl of samples, 50 μl of calibration levels and 50 μl of quality control in a 96-deepwell plate, and then centrifuged (4°C, 30 min, 3000 rpm). Calibration levels and quality controls were prepared in PBS containing BSA (4% w/v). 175 μl of supernatant were transferred to a 96-deepwell plate and evaporated under a nitrogen stream at 40°C for 30 min. The dried residue was reconstituted with 100 μl of MeOH/H_2_O (1:9) solution and then centrifuged (4°C, 10 min, 3,000 rpm). 80 μl of each sample was then transferred to a 98-deepwell plate, and 5 μl were injected into the LC-MS/MS system. For brain and liver samples, the tissues were freeze-dried for 48 h. Each dried sample was then weighed between 2.5 and 3 mg. Each weighed sample was homogenized with 250 μl of MeOH/H_2_O (1:1) and a glass bead using a Precellys homogenizer. The homogenate was subjected to 30 min of planar agitation and subsequently centrifuged (4°C, 15,000g, 10 min). Supernatants (150 μl) were transferred into another tube for the quantification process. For LC-MS/MS quantification, calibration curve and quality controls were prepared in milliQ water. In a 96-deepwell plate, a 50 μl aliquot was taken for the calibration curve and for the quality control samples, as well as 50 μl of each tissue extract supernatant, and 50 μl of internal standard solution. The plate was shaken on a planar shaker for 5 min. The injection volume was set to 15 μl into the LC-MS/MS system.

LC was performed using an UPLC Waters® in Reversed Phase, on a C18-XB 1.7 μm 100 Å 150 × 2.1 mm column eluted with solvent A: H_2_O + 0.5% formic acid and solvent B: methanol + 0.5% formic acid at a flow of 0.4 ml/min. MS parameters were: Spectrometer: Waters QqQ with ESI with Multiple Reaction Monitoring (MRM) in negative and positive mode. For data treatment, a TargetLynx Waters® software was used. A calibration curve for each metabolite was created by calculating the intensity ratio between the metabolite and its internal standard. Each calibration curve is characterized by a quantification range from the Lower Limit of Quantification (LLOQ) to the Upper Limit of Quantification (ULOQ). The analytes, along with their LLOQ and ULOQ values, are detailed in Supplemental Tables S3 and S4. Quality Control was used to validate the quantification batches. The « %Diff » formula was used to assess the difference between the calculated and the theorical concentrations: %Diff = 100 – [(Experimental concentration – Theorical concentration)/Theorical concentration] ∗100. Other mass spectrometric parameters, such as parent and fragmentation ions, collision energy etc., are available from ref. (45) and Supplementary materials therein.

For univariate analysis, statistical analysis was performed using MetaboAnalyst (46).

## Results

### Effect of colonization of GF mice with *E. coli* strains on intestinal parameters

**Fig. 1:**
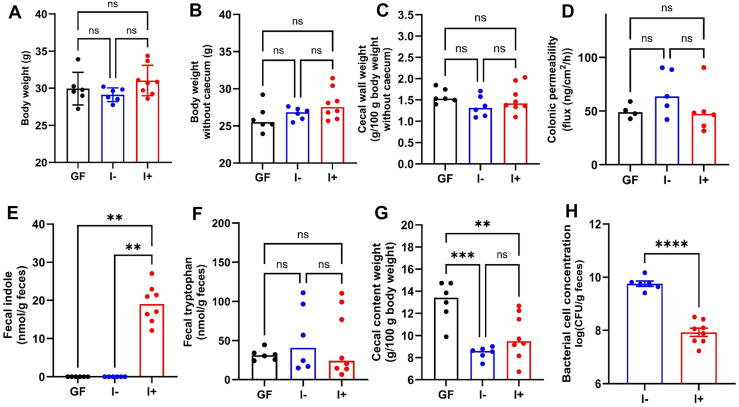
Effects of *E. coli* strain colonization on intestinal parameters in GF mice. Bar graphs illustrate (A) Body weight, (B) Body weight without caecum (g), (C) Cecal wall weight (g/100g body weight, excluding cecum content), (D) Colonic permeability (flux(ng/cm2/h)), (E) Fecal indole levels (nmol/g feces), (F) Fecal tryptophan levels (nmol/g feces) (G) Cecal content weight (g/100g body weight), (H) Bacterial cell concentration (log CFU/g feces). Data are presented as mean ± SD. Statistical significance was determined using one-way ANOVA followed by Tukey's post hoc test or Kruskal-Wallis followed by Dunn's test. ∗*P* < 0.05, ∗∗*P* < 0.01, ∗∗∗*P* < 0.001, ∗∗∗∗*P* < 0.0001.

Colonization of GF mice with I+ or I− strains of *E. coli* produced very similar effects on body weight, with or without cecum (Fig. 1A, B), cecum wall weight (Fig. 1C), and colonic permeability (Fig. 1D). As expected, colonization with the I+, but not the I−, strain resulted in very high levels of indole in the cecum (Fig. 1E), although the levels of tryptophan (Fig. 1F) were similar for the two strains. While cecal content weight was similar again after colonization with either strain, and significantly lower than in uncolonized GF mice (Fig. 1G), the amount of bacterial cell concentration in feces was significantly higher with the I− than the I+ strain (Fig. 1H).

### Effect of GF mouse colonization with *E. coli* strains on brain NAE and 2-MAG levels

We analyzed selected eCBome mediators, namely the NAEs and 2-MAGs, which are the respective congeners of AEA and 2-AG, in brain areas involved in the control of emotional behavior.

**Fig. 2:**
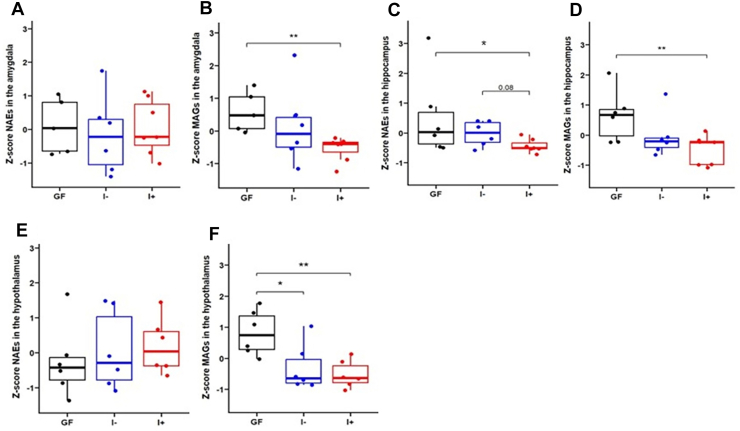
Z-scores of NAEs and MAGs in the brain. Boxplots illustrate (A) Z-score for NAEs and (B) MAGs the amygdala, (C) Z-score for NAEs and (D) MAGs in the hippocampus, (E) Z-score for NAEs and (F) MAGs in the hypothalamus. Data are presented as median and interquartile range. Statistical significance was determined using Kruskal-Wallis followed by Dunn’s post hoc test. ∗*P* < 0.05, ∗∗*P* < 0.01, ∗∗∗*P* < 0.001, ∗∗∗∗*P* < 0.0001.

In the amygdala, a general statistically significant decrease in 2-MAGs, but not NAEs, was observed following colonization only with the I+ strain (Z-score, *P* = 0.009) (Fig. 2A). In particular, this decrease seemed to be driven by the decrease in 2-AG (only with the Kruskal–Wallis test) and 2-oleoyl-glycerol (2-OG, with both tests used) (Supplemental Fig. S2A).

In the hippocampus, both 2-MAGs and NAEs were decreased following colonization, only with the I+ strain (Fig. 2C, D) (Z-score MAGs, *P* = 0.022, Z-score NAEs, *P* = 0.041). The effect on 2-MAGs was driven by the strongly significant decrease of 2-OG and by trends in the decrease of other 2-MAGs and occurred despite a significant increase in 2-palmitoyl-glycerol (2-PG) levels (Supplemental Fig. S2B). In the case of NAEs, the effect was instead driven by a strongly significant decrease of *N*-linoleoyl-ethanolamine (LEA, with both tests), and by trends for the decrease of *N*-oleoyl-ethanolamine (OEA) and *N*-palmitoyl-ethanolamine (PEA), while AEA levels were significantly higher with the I+ strain only when compared to the I− strain (with both tests) (Supplemental Fig. S2B).

In the hypothalamus, indole-independent effects were noted on 2-MAGs, which were in fact decreased following colonization with both I+ and I− strains (Z-score, *P* = 0.001, 0.013) (Fig. 2F). These effects seemed to be driven by statistically significant effects on several 2-MAG species, including 2-AG, and observed with both statistical tests used (Supplemental Fig. S2C).

In the striatum, nucleus accumbens and prefrontal cortex, no statistically significant effect was observed overall on either 2-MAGs or NAEs, nor on single members of these two families of eCBome mediators (data not shown).

### Effect of GF mouse colonization with *E. coli* strains on NAE and 2-MAG levels

**Fig. 3:**
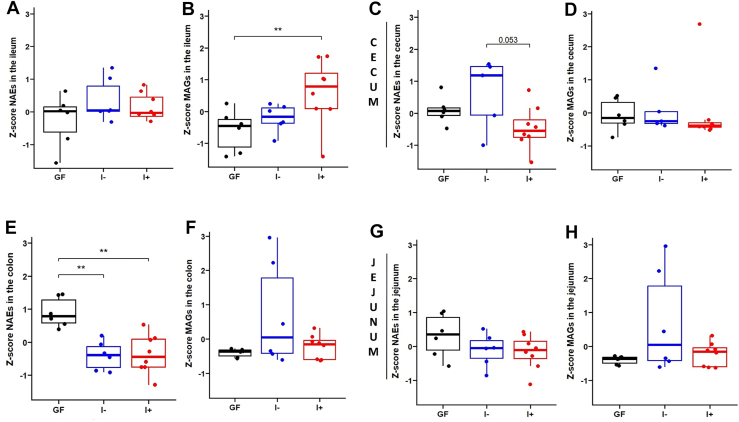
Z-scores of NAEs and MAGs in the intestine. Boxplots illustrate (A) Z-score for NAEs and (B) MAGs in the ileum, (C) Z-score for NAEs and (D) MAGs in the cecum, (E) Z-score for NAEs and (F) MAGs in the colon, (G) Z-score for NAEs and (H) MAGs in the jejunum. Data are presented as median and interquartile range. Statistical significance was determined using Kruskal-Wallis followed by Dunn’s post hoc test. ∗*P* < 0.05, ∗∗*P* < 0.01, ∗∗∗*P* < 0.001, ∗∗∗∗*P* < 0.0001.

In the ileum of GF mice colonized with the I+, but not the I−, strain, higher levels of 2-MAGs (Z-score, *P* = 0.01) were observed (Fig. 3A, B) that were likely driven by increases in 2-PG, 2-OG and several polyunsaturated members of this family of lipids, although the effect for the eCB 2-AG was not statistically significant (Supplemental Fig. S3A). No effect on NAEs was observed instead, except for an increase in *N*-docosahexaenoylethanolamine (DHEA) with the I− strain.

In the cecum, NAE levels following colonization with the I+ strain were significantly lower (Z-score, *P* = 0.053) than those in mice colonized with the I− strain, but not than those in GF mice (Fig. 3C, D). This effect was due to LEA, PEA and *N*-stearoyl-ethanolamine (SEA). However, the cecum levels of DHEA and OEA in I+, but not I−, -colonized mice were significantly lower than those of GF mice (Supplemental Fig. S3B). Furthermore, while no overall significant effect on 2-MAGs was observed (Z-score, *P* = 0.32), some members of this family of lipids were either higher than in GF mice (i.e. 2-OG), or lower than in mice colonized with the I− strain (i.e. 2-linoleoyl-glycerol, 2-LG) (Supplemental Fig. S3B).

In the colon, we observed a generalized reduction in NAE levels (Z-score, *P* = 0.002, 0.003) of both I− and I+ colonized mice (Fig. 3E, F). This was driven, in manners that were either statistically significant with one or both of the two used tests, by all NAEs, although the effects on LEA, *N*-eicosapentaenoyl-ethanolamine (EPEA) and *N*-docosapentaenoyl-ethanolamine n-3 (DPEA n-3) were significant and strong only with the I+ strain (Supplemental Fig. 3C). Regarding 2-MAGs, although not statistically significant for the overall family of lipids when using the Z-score (*P* = 0.58), several individual congeners of 2-AG were significantly higher either only in mice colonized with the I+ strain (i.e. 2-LG and 2-OG) or in mice colonized with the I− strain (2-PG), and trends for effects similar to either scenario were seen with other 2-MAG species (Supplemental Fig. S3C).

Finally, while in the duodenum no effect whatsoever was noted (data not shown), in the jejenum non-statistically significant effects were observed in mice colonized with either strain (Fig. 3G, H). When looking at each single member of the two families of lipids, jejunum levels of PEA, OEA, DPEA n-6 were decreased, and of 2-OG and 2-LG were increased, in mice colonized with either strain (Supplemental Fig. S3D).

### Effect of colonization with *E. coli* strains on GF mouse brain eCBome receptor and metabolic enzyme expression

For these analyses, we focused our attention on the two brain areas, that is, the amygdala and the hippocampus, and observed indole production-dependent changes in eCBome mediators, that is, in NAE and 2-MAG levels (Fig. 2).

**Fig. 4:**
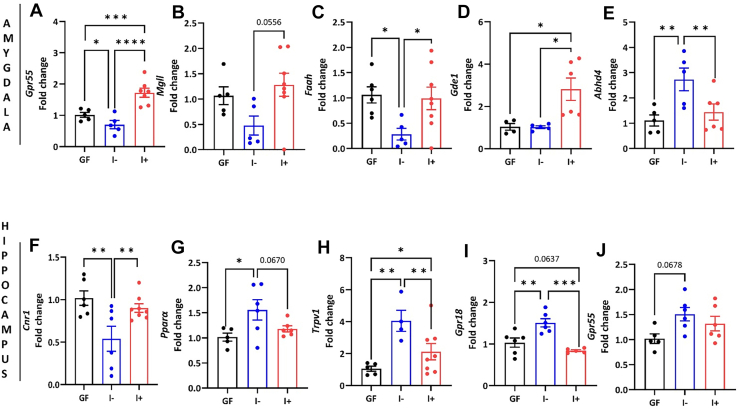
Gene expression of the eCBome in the brain. Bar graphs illustrate (A–E) mRNA expression of Gpr55, Gde1, Faah, Mgll, Abhd4 in the amygdala. (F–J) mRNA expression of *Cnr1*, *Ppara*, *Trpv1*, *Gpr18*, and *Gpr55* in the hippocampus. Data are presented as mean ± SD. Statistical significance was determined using one-way ANOVA followed by Tukey's post hoc test. ∗*P* < 0.05, ∗∗*P* < 0.01, ∗∗∗*P* < 0.001, ∗∗∗∗*P* < 0.0001.

In the amygdala, a strong increase in the mRNA expression of *Gpr55* (Fig. 4A), a receptor for several NAEs, and of *Gde1* (Fig. 4B), a biosynthetic enzyme for NAEs, was noted following colonization with the I+, but not the I−, strain. Conversely, colonization with the I−, but not the I+, strain caused a reduction in the mRNA expression of NAE-hydrolyzing enzyme, *Faah* (Fig. 4C), as well as for the 2-MAG-hydrolyzing enzyme *Magl* (as a trend) (Fig. 4D), and a significant increase, instead, for the NAE-biosynthesizing enzyme *Abhd4* (Fig. 4E). Other genes expressing several receptors and metabolic enzymes for NAEs and 2-MAGs were not altered (Supplemental Fig. 4A).

In the hippocampus, only colonization with the I− strain caused some statistically significant alterations, in particular with a decrease of *Cnr1* mRNA expression (Fig. 4F) and an increase in several other receptors, that is, *Pparα*, *Trpv1*, *Gpr18* and *Gpr55* (Fig. 4G–J). The only exception was *Trpv1*, whose expression was slightly increased also by the I+ strain, but significantly less than with the I− strain (Fig. 4B). No effect was observed on the expression of eCBome metabolic enzymes in this brain area (Supplemental Fig. S4B).

### Effect of colonization of GF mice with *E. coli* strains on intestinal eCBome receptor and metabolic enzyme expression

We will describe here only changes that were statistically significant or nearly significant. Other data are described in Supplemental Fig. S5.

**Fig. 5:**
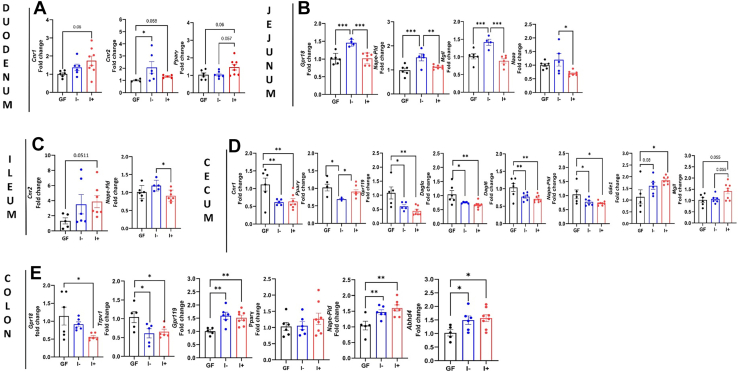
Gene expression of the eCBome in the intestine. Bar graphs illustrate (A) mRNA expression of *Cnr1*, *Cnr2*, *Pparγ* in the duodenum; (B) mRNA expression of *Gpr18*, *Nape-Pld*, *Mgll*, *Naaa* in the jejunum; (C) mRNA expression of *Gpr18* and *Nape-Pld* in the ileum; (D) mRNA expression of *Cnr1*, *Gpr119*, *Pparγ*, *Daglα*, *Daglβ*, *Nape-pld*, *Gde1*, *Mgll*, in the cecum; (E) mRNA expression of *Cnr2*, *Trpv1*, *Gpr119*, *Pparγ*, *Nape-Ppld*, *Abhd4*, in the colon. Data are presented as mean ± SD. Statistical significance was determined using one-way ANOVA followed by Tukey's post hoc test. ∗*P* < 0.05, ∗∗*P* < 0.01, ∗∗∗*P* < 0.001, ∗∗∗∗*P* < 0.0001.

In the duodenum, nearly statistically significant increases were seen for *Cnr1* and *Pparγ* mRNA expression following colonization with the I+ strain, whereas *Cnr2* mRNA expression was increased by both the I− and, as a trend, the I+ strain (Fig. 5A).

In the jejunum, most changes were observed only following colonization with the I− strain, i.e. increases for the *Gpr18* receptor, the *Nape-Pld* enzyme for NAE biosynthesis, and the MAGL enzyme for 2-MAG degradation. The NAE-hydrolyzing enzyme *Naaa* was decreased in I+ as compared to I− -colonized mice (Fig. 5B).

In the ileum, an interesting effect of I+ colonization was the significant decrease in the mRNA expression of *Gpr18*. *Nape-Pld* expression was instead reduced in I+ as compared to I− -colonized, but not to uncolonized GF, mice (Fig. 5C).

In the cecum, most of the changes were observed after both I+ and I− strain colonization of GF mice. In particular, the mRNA expression of *Cnr1* and *Gpr119* receptors was decreased, as was that of the 2-MAG biosynthesizing enzymes *Daglα* and *Daglβ*, of *Nape-Pld* and of *Naaa* (Fig. 5D). However, the expression of some genes was significantly altered only by the I+ strain, as in the case of the increases of *Gde1* and *Magl* mRNAs, or the I− strain, as in the case of the decrease of *Pparγ* mRNA (Fig. 5D).

Finally, also in the colon, most of the significant alterations were observed with both I+ and I− strain colonization of GF mice. This was the case of the decrease of *Trpv1*, and the increase of *Gpr119*, mRNA, on the receptor side, and of the increases of *Nape-Pld* and *Abhd4* mRNAs, on the enzyme side. However, in this intestinal section also some significant changes were seen only with the I+ strain, as with the increase of *Cnr2* (though a similar trend is observed with the I− strain), and the trend of increase for *Pparγ* mRNA (Fig. 5E).

### Effect of colonization with *E. coli* strains on GF mouse plasma NAEs and 2-MAGs levels

**Fig. 6:**
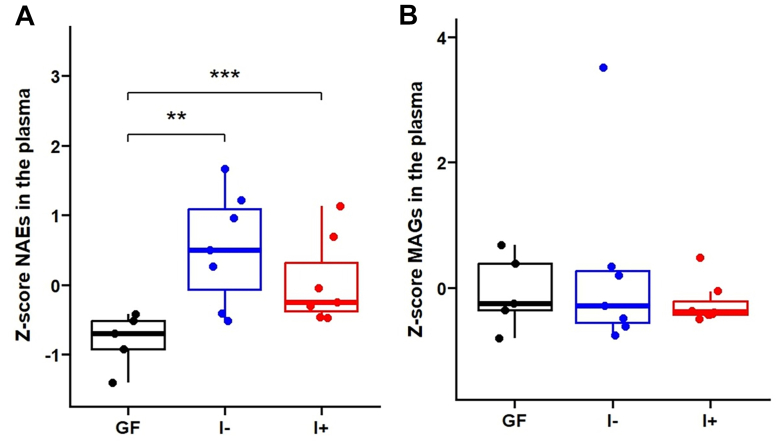
Z-scores of NAEs and MAGs in the plasma. Boxplots illustrate (A) Z-score for NAEs and (B) MAGs in the plasma. Data are presented as median and interquartile range. Statistical significance was determined using Kruskal-Wallis followed by Dunn’s post hoc test. ∗*P* < 0.05, ∗∗*P* < 0.01, ∗∗∗*P* < 0.001, ∗∗∗∗*P* < 0.0001.

Colonization with either I+ and I− strains increased overall plasma NAE levels (Z-score, *P* = 0.003, *P* = 0.025), without altering 2-MAG concentrations (Z-score, *P* = 0.63, *P* = 0.49) (Fig. 6). At the level of the single NAEs, OEA, LEA and DHEA were elevated with both strains, whereas AEA, PEA, SEA, DPEA n-3 and DPEA n-6 were significantly elevated only with the I− strain, which also reduced 2-docosapentaenoyl-glycerol (2-DPG) (n-3) (Supplemental Fig. S6).

### Effect of colonization with *E. coli* strains on GF mouse plasma, liver and brain indole and tryptophan metabolites

**Fig. 7:**
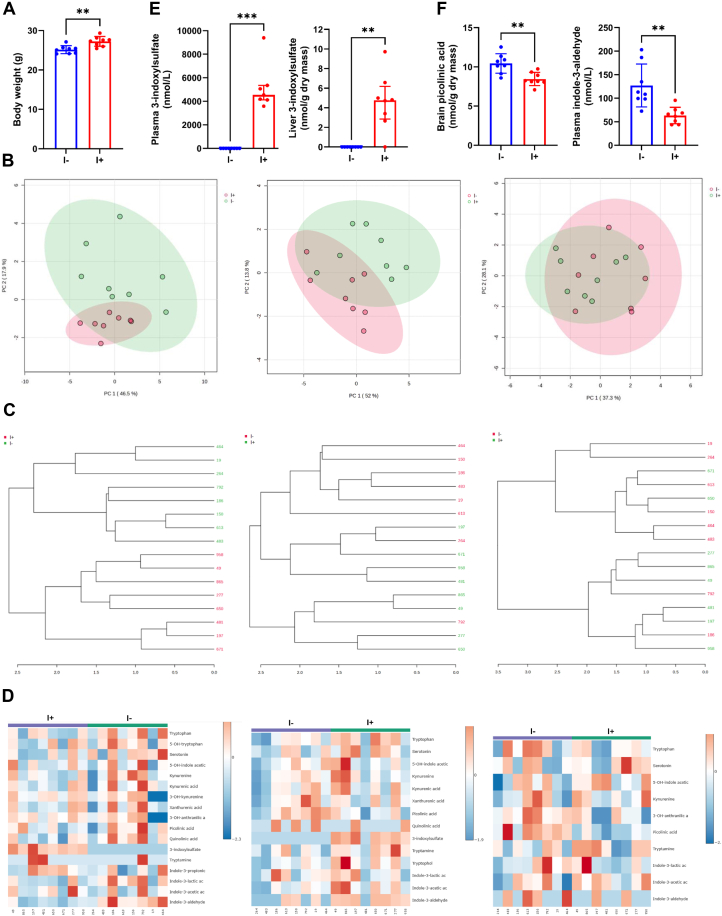
Targeted metabolomics analysis of tryptophan metabolites. (A): Body weight (g) of the I− and I+ mice of the cohort used for targeted metabolomics analysis of tryptophan metabolites. Data are presented as mean ± SD. Statistical significance was determined using unpaired Student’s *t* test. ∗∗*P* < 0.01. (B): Scores plots of the PCA analyses of the plasma (left panel), liver (central panel), and brain (right panel) profiles in the I− and I+ mice of the cohort used for this analysis are shown. Plasma and liver data were normalized using square-root transformation; this treatment was not necessary for brain data. Autoscaling was applied to all the data. I− and I+ mice cluster separately based on their plasma (Permanova F-value: 3.452; R-squared: 0.1978; *P*-value [based on 999 permutations]: 0.05) and liver (Permanova F-value: 4.0034; R-squared: 0.22237; *P*-value [based on 999 permutations]: 0.044) profiles, but not based on their brain profiles (Permanova F-value: 1.8919; R-squared: 0.11905; *P*-value [based on 999 permutations]: 0.171). Metabolites that were below the lower limit of quantification in all the samples of a given tissue were not included in the analysis. (C): Dendrograms showing that I− and I+ mice in the cohort used for this analysis cluster separately based on their plasma (left panel) and liver (central panel), but not their brain (right panel), profiles. Plasma and liver data were normalized using square-root transformation; this treatment was not necessary for brain data. Autoscaling was applied to all the data. Distance measure: Pearson; clustering method: average. Metabolites that were below the lower limit of quantification in all the samples of a given tissue were not included in the analysis. (D): Heatmaps showing the relative concentrations of the metabolites in the plasma (left panel), liver (central panel) and brain (right panel) of I− and I+ mice of the cohort used for this analysis. Plasma and liver data were normalized using square-root transformation; this treatment was not necessary for brain data. Autoscaling was applied to all the data. Distance measure: Pearson; clustering method: average. Metabolites that were below the lower limit of quantification in all the samples of a given tissue were not included in the analysis. (E): Concentrations of indoxyl-3-sulfate in the plasma (left panel) and liver (right panel) in the I− and I+ mice of the cohort used for targeted metabolomics analysis of tryptophan metabolites. Data are presented as medians with interquartile range. Statistical significance was determined using a Mann-Whitney test. ∗∗*P* < 0.01, ∗∗∗*P* < 0.001. Indoxyl-3-sulfate in the brain was below the lower limit of quantification in both I− and I+ mice. (F): Concentrations of picolinic acid in the brain (left panel) and indole-3-aldehyde in the plasma (right panel) in the I− and I+ mice of the cohort used for targeted metabolomics analysis of tryptophan metabolites. Data are presented as mean ± SD. Statistical significance was determined using unpaired Student’s *t* test (with Welch correction for unequal SDs for plasma indole-3-aldehyde data). ∗∗*P* < 0.01.

A separate cohort of I− and I+ *E. coli*-colonized GF mice was used for this study, with the aim of checking if indole metabolites were present in peripheral and central tissues of colonized mice. The liver was used as a proxy of a target peripheral organ following indole absorption from the intestine. Overall, there was no difference in fecal bacterial concentration, cecal wall weight, and cecal content weight between the two groups of mice. However, I− colonized mice showed a slightly lower body weight in this experiment (Fig. 7A). On the other hand, PCA plots, dendrograms and heatmaps show that the plasma and liver metabolomes of I+ mice cluster separately from those of the I− mice (Fig. 7B–D), this separation being driven mainly by the differences in indoxyl-3-sulfate, which in fact was significantly higher in the plasma and liver of I+ versus I− -colonized mice (Fig. 7E). Importantly, no separate clustering for the metabolome, nor any presence of indoxyl-3-sulfate, was observed in the brain of the two groups of mice (Fig. 7B–E). Other small differences were noted, i.e. lower picolinic acid levels in the brain and lower indole-3-aldehyde levels in the plasma of I+ colonized mice (Fig. 7F).

## Discussion

In this study we have provided unprecedented data showing that: 1) colonization of GF mice with *E. coli* strains affects the gut and brain eCBome, and in particular NAE and 2-MAG signaling; and 2) different effects on the eCBome signaling system are observed depending on whether or not the colonizing *E. coli* strain has the capability of producing indole. These data strengthen previous reports suggesting that gut microbiota regulate eCBome signaling and point to the production of indole from tryptophan metabolism as a novel mechanism through which this regulation can occur. Additionally, in view of the well-known effects of eCBs and eCB-like mediators on emotional behaviors, these results suggest that the previously reported effects of indole on emotionality (i.e. increased anxiety- and depressive-like behaviors) in mice and rats (9, 10, 11) may be partly due to capability of this metabolite to impact the eCBome signaling-mediated gut-brain axis. Finally, it is intriguing to speculate, based on these results, that the eCBome may play also a role in the described effects of indole on intestinal functions, which are variedly affected also by eCBome mediators and receptors, such as the regulation of epithelial cell tight junction and intestinal permeability, or the release of GLP-1 from enteroendocrine L-cells and the subsequent control of appetite and insulin secretion (47).

**Fig. 8:**
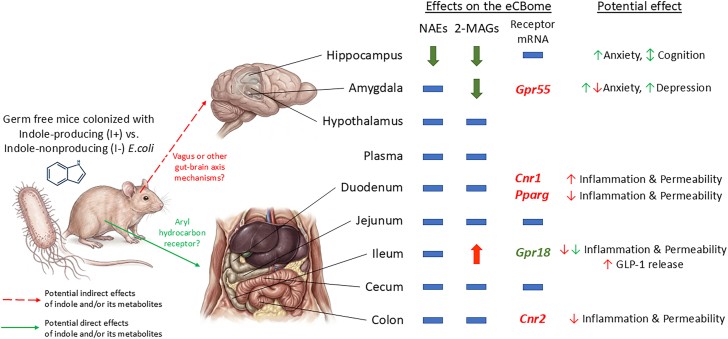
Schematic representation of the major findings of this study and potential mechanisms and physiopathological consequences of the observed eCBome alterations following I+ *E. coli* colonization. Specific effects of I+ *E. coli* colonization of germ-free mice are shown for brain and intestinal tissues because of potential indirect or direct effects, respectively, hypothesized based on the results of the metabolomics analyses described in Figure 7 and the finding of indoxyl-3-sulphate only in the liver and plasma. The potential final effect in terms of modulation of affective behavior and intestinal functions (inflammation or permeability) are depicted with arrows of the same color as those used to indicate the respective change in eCBome signaling in the various tissues (downward arrows= decrease; upward arrows= increase).

Among the indole-induced alterations that we observed here, the most worth mentioning are the effects in the amygdala, a brain region strongly involved in mood regulation, where gut colonization with the I+, but not the I−, *E. coli* strain caused a reduction in the concentrations of the eCB, 2-AG, as well as of 2-OG, and an increase of the mRNA expression of the AEA and PEA receptor, *Gpr55*. Reduced brain levels of 2-AG and other 2-MAGs in connection with alterations in the composition of gut microbiota have been previously reported in a UCMS model of depression and shown to mediate the triggering effect of chronic stress on depressive-like behaviors in this model (34). Therefore, it is tempting to speculate that the similar effect of I+ *E. coli* in UCMS-induced emotional disturbance in mice (10) is also due to its inhibition of 2-AG signaling in the amygdala, as well as of 2-MAG levels in the hippocampus, observed here. The increase in the amygdala of the mRNA expression of *Gpr55* as well as of the NAE-biosynthesizing enzyme *Gde1* (although not resulting in enhanced amygdalar NAE levels under our experimental conditions) by I+ *E. coli* may instead provide negative feedback protection against indole-induced anxiety, since activation of this receptor (and some NAEs are indeed agonists for GPR55) has been shown previously to produce anxiolytic actions in several mouse models (48, 49, 50). Other eCBome mediators specifically affected in the brain by I+ *E.coli* in our study were endogenous PPARα agonists, that is, the NAEs, LEA, OEA and PEA, which were (or strongly tended to be) reduced in the hippocampus following colonization with the I+ strain. Anxiolytic and anti-depressive actions of endogenous agonists of PPARα, including PEA, have been reported in rodents (51, 52, 53), and therefore, this effect of indole may again contribute to its negative actions on emotionality in GF mice. On the other hand, we also found here that another endogenous PPARα agonist, 2-PG (54), was instead increased in the hippocampus following colonization with the I+ strain. Interestingly, the hippocampus is the brain region where it has been found that the indole metabolite, isatin, is present at the highest concentrations in rats (55), and the other indole metabolite, oxindole, decreases neuronal excitability by increasing the threshold and latency of action potentials (56), pointing to the importance of this area in the brain circuitries regulated by these tryptophan metabolites (but see also below the discussion of the results of Fig. 7). Figure 8 schematically depicts some potential eCBome-based mechanisms underlying I+ *E. coli* actions in the brain that may emerge from our present study and will need to be addressed experimentally in the future.

I+ strain-induced changes in eCBome signaling were also observed in the intestinal tract (Fig. 8). Worth mentioning are the increases in 2-MAGs (although not 2-AG) in jejunum, ileum, cecum and colon, which may be seen as being in contrast with the findings of Chevalier *et al.* (34), who reported a decrease of 2-MAGs in the intestine of mice with UCMS-induced depression-like behaviors and gut microbiota alterations. This suggests that while, in our previous study in GF mice (10) indole might have contributed to the effects of UCMS on emotionality via the disruption of 2-MAG signaling in the brain, this is clearly not the only pathway underlying chronic stress-induced depressive-like behaviors. Indeed, we previously demonstrated that indole, when administered directly in the cecum, induces the activation of the vagus nerve and increases the brain levels of oxindole and isatin (9), two indole metabolites known for their effects on rodent behaviors (9, 10, 57, 58). More recently, we confirmed the implication of the vagus nerve and also the stimulation of the dorsal motor nucleus of the vagus and demonstrated the role of norepinephrine neurons of the locus coeruleus, in the mechanism of action of indole when gastro-intestinally administered. This effect was also associated with anxiety-like behaviors (11). We found here that several NAEs were decreased specifically by the I+ strain in the cecum and, limited to some members of this family, the colon. In any case, to what extent specific changes in intestinal 2-MAG and NAE levels may have contributed to the behavioral effects of indole in GF mice is difficult to say, since the role of the gut eCBome in the control of affective behaviors is still poorly understood. Guida *et al.* showed that gut microbiota alterations induced by antibiotic treatment lead to small intestinal eCBome changes associated with hippocampal neuroglial reorganization and depressive-like behaviors in mice (59), supporting the notion of the importance of the gut microbiome-eCBome axis in gut-brain communication in the context of affective disorders. More specifically, the presence of eCBome receptors, such as CB1 and TRP channels of vanilloid type-1 (TRPV1), in myenteric and vagal fibers highlights the potential impact of gut-produced 2-MAGs and NAEs on brain function (60). Furthermore, the fact that indole also acts through the activation of the vagus nerve generates new questions concerning the interactions between this tryptophan metabolite and eCBome signaling. We also previously demonstrated that gut microbiota could modulate the gut as well as the brain eCBome (21, 22), and thus the implication of intestinal 2-MAGs and NAEs in the effects of commensal microorganisms on affective disorders is certainly worth exploring. Interestingly, at least in the cecum, the increased levels of 2-MAGs observed here cannot be explained by altered mRNA expression of the 2-MAG degrading enzyme, *Mgll*, which was also increased in this intestinal section. Instead the increase in NAEs levels in the cecum may be partly explained by increased mRNA expression of the NAE-biosynthesizing enzyme *Gde1* (see also below).

Here we found little changes in the expression in the intestine of eCBome receptors for 2-MAGs and NAEs, since mRNA levels of *Pparα*, *Gpr119* and *Trpv1*, which are the best-known molecular targets of non-eCB eCBome mediators, were not altered specifically by the I+ strain. Instead, indole seemed to alter the expression of genes that encode for receptors, such as *Cnr1* (increased in the duodenum), *Cnr2* (increased in the colon) and *Pparγ* (increased in both the duodenum and colon) that are exclusively activated by eCBs (i.e. AEA for all three receptors, and 2-AG for CB1 and CB2) and not by other eCBome mediators. An exception might be represented by the decrease of *Gpr18* expression observed in the ileum, as this receptor has also been suggested to be activated by both AEA and other *N*-acylamines, and its role in emotional behaviors has been investigated in a recent study, which highlighted beneficial actions similar to those of CB1 activation (61). Future investigations on the bidirectional cross-talk between indole and the intestinal eCBome and its role not only in behavior but also in intestinal function (Fig. 8) will allow for a better understanding of eCBome-mediated control of the gut-brain axis.

In several brain areas and intestinal sections, colonization with either the I+ or I− strain produced the same effect on some eCBome mediators, metabolic enzymes or receptors. This phenomenon, which is clearly not linked to the production of indole by *E. coli*, might be instead related to the general effect of gut microorganisms on the eCBome in GF mice previously reported by us (21, 22, 62). Indeed, we previously showed that reintroduction, through a FMT, of gut microbiota into GF mice, reduced NAE levels and *Pparγ* and *Cnr1* expression, while elevating *Gpr18* and *Gpr55* expression, in the gut, (21) and elevated both NAE and 2-MAG levels in the brain (22). Accordingly, here we found *E. coli* colonization reduced NAE levels in the colon and cecum, and *Cnr1* expression in the cecum. However, other previously observed FMT-induced effects were not reproduced here by *E. coli* colonization, thus reinforcing the concept of the multiplicity of gut microbiota regulation of the eCBome, exemplified also by the fact that other commensal bacteria-derived metabolites, as well as several probiotic strains, can alter this signaling system (12, 21, 54, 63).

Finally, several effects were also noted on eCBome mediators, receptors and metabolic enzymes only with the I− strain. These effects could be explained, at least in part, by the fact that colonization with this strain resulted in a gut bacterial cell population significantly higher than that observed with the I+ strain, as shown by the higher bacterial cell concentration measured in the feces. This latter effect may in turn be explained by the previously described inhibitory effect of indole on *E. coli* division (64), or on the expression of *E. coli* structural proteins (as exemplified by the previously reported effects on *Flir*, coding for the flagellar biosynthetic protein, or *FimZ*, coding for Fimbiral protein Z) (65). In two cases, however, the two strains produced opposite effects on eCBome components with respect to uncolonized GF mice: on *Gpr55* expression in the amygdala (decreased by I− and increased by I+), and on LEA levels in the cecum (increased by I− and decreased by I+). This might indicate the existence of other metabolites in *E. coli* that oppose some of the effects of indole on these two specific eCBome components. A full metabolomics study of these strains would be needed to investigate these possibilities.

Several indole-dependent and independent changes in metabolic enzyme expression were noted in this study that, in most cases, did not seem to correlate with corresponding changes in NAE and 2-MAG levels. This is not unusual, as a similar phenomenon was also noted in many previous studies on eCBome signaling, including our previous ones in GF mice with and without FMT (21, 22). The discrepancy between NAE/2-MAG tissue levels and metabolic enzyme expression might be due, among others, to the fact that biosynthetic precursor availability, rather than enzyme expression, seems to play a key rate-limiting role in the regulation of the concentrations of these mediators.

Plasma levels of NAEs and 2-MAGs were not specifically modulated by the I+ strain and seemed to be more under the influence of *E. coli* colonization regardless of its capability of producing indole. This finding reinforces the concept that the circulating levels of these mediators do not reflect their intestinal (and brain) levels through spillover from these organs, and are in agreement with our previous findings in a human cohort (66).

The possibility exists that the effects observed following colonization with the I + *E. coli* strain were not due directly to indole but to the fact that this metabolite, or the lack thereof, changes the biology of *E. coli* and its capability of producing other metabolites affecting the eCBome. Therefore, we sought to obtain some preliminary information on whether indole coming from colonization with the I+ strain was indeed reaching, and being metabolized by, the host by analyzing GF mouse plasma, liver and brain for the presence of tryptophan metabolites using LC/MS-MS. The data obtained indicate that the major indole metabolite, indoxyl-3-sulfate, can be found in significantly higher levels in the I+ as compared to the I− *E. coli*-colonized mice, though only in the plasma and liver. This is suggestive of the fact that indole produced by *E. coli* does reach the host and is metabolized by it, though it does not reach the brain as efficiently as it reaches peripheral tissues under our experimental conditions. This finding, in turn, suggests that indole effects on the brain eCBome may be due to indirect mechanisms starting in the gut or other peripheral organs, such as, for example, through the interaction with the gut mucosa or the enteric nervous system, vagal nerve-mediated pathways or via second messengers from the gut immune system or enteroendocrine cells, rather than through indole and its metabolites reaching the brain. It is also possible that indole and/or indoxyl-3-sulfate are responsible for the effects of indole in the periphery and that other metabolites that we did not find here to be increased only in I+ *E. coli* colonized mice are instead responsible for effects in the brain. Indeed, we could not analyze in this study minor indole derivatives, such as oxindole or isatin, which are very difficult to track down with our current methodology. However, as mentioned above, it was previously reported that these metabolites are increased in the cecum following indole administration (9). As for other tryptophan metabolites, we detected reduced picolinic acid levels in the brain and indole-3-aldehyde levels in the plasma, which may be suggestive of how I+ *E. coli* may indirectly or directly affect other pathways of tryptophan metabolism in the host. Future studies should investigate whether these two metabolites play any role in I+ *E. coli* effects on the eCBome.

Given the variability in the measured levels of some of the single eCBome mediators, which was one of the reasons why we used the Z-score applied to the whole families of NAEs and 2-MAGs to assess differences among groups, and, in some cases, both parametric and non-parametric tests to assess statistically significant differences, one limitation of this study is that it may have been somewhat underpowered. Additionally, eCBome measurements were carried out only in one cohort of mice, although the efficacy of the colonization was reproduced also in the cohort used for metabolomics analyses. All this may have weakened some of the conclusions drawn from the results.

In conclusion, we have provided here data suggesting that indole production by gut bacteria, as observed previously also with short-chain fatty acids (23, 24), may be one of the key mechanisms by which gut bacteria modulate intestinal and brain eCBome signaling. Future studies will now need to address the issue of the molecular mechanisms by which this important tryptophan metabolite, or some of its catabolic products, alter the levels of NAEs and 2-MAGs and the mRNA expression of their receptors and metabolic enzymes, and if these alterations explain part of its effects on emotionality in mice (10, 11).

## Data availability

All data supporting the findings presented in this study are available within the article or accompanying materials. Raw data are available from the corresponding author upon reasonable request.

## Supplemental data

This article contains supplemental data.

## Conflict of interest

The authors declare that they have no conflicts of interest with the contents of this article.
