# Supplementary material for: Intestinal colonization of germ-free mice with indole-producing E. coli modulates the central and peripheral endocannabinoidome
Source: J Lipid Res. 2026 May 26;67(7):101070. doi: 10.1016/j.jlr.2026.101070 (PMC13320190; doi:10.1016/j.jlr.2026.101070)
Supplement: Supplementary Material [file mmc1.docx]

**SUPPLEMENTARY INFORMATION**

**Suppl. Table 1:**

List of primers used in RT-PCR analysis

| Gene name | Forward | Reverse |
| --- | --- | --- |
| *Abdh4* | ATGGCTGATGATCTGGAGCAG | CGGGCCAGGAACTTGTTCT |
| *Abdh6* | CAATCCTGGCATTTGTTGCGT | ATGGTGTGCGTAGCGAACTT |
| *Cnr1* | AATGCCTTAGAACTGGATGACA | TGCAACTTCTCAATGTAGCCT |
| *Cnr2* | GCTCTTGGGACCTACGTG | GCTGCGATTTTGTATTCCTCTG |
| *Dagl*α | TCTTCGGCTTGGTCTATAACCC | TCGGCAATCATACAGCTCAGA |
| *Dagl*β | GGGCAAGGCGGCTCAAGTGT | GCCTCACAGAAGCCACGCACA |
| *Faah* | ACTTGGACGTGGTGCTAACC | GCCTATACCCTTTTTCATGCCC |
| *Gde1* | GCTGCGGTTCTTCAGCTTC | CTCCACACCTGTTGCTCCATT |
| *Gpr18* | CACCCTGAGCAATCACAACCA | AGTGACATTAACAAACAGCCCA |
| *Gpr55* | CACTAAGGGCTGGGTACAAAAG | GCGGTTCCTCACCAGATACTG |
| *Gpr119* | CTTGCTGTCCTAACCATCCTCA | CCACGCCAATCAAGGTATCAG |
| *Mgll* | GGAAGCCCAGTGGCACACCC | CACAGCAAACGCCTCGGGGA |
| *Naaa* | CCTGGCTGACGGTATCTTGG | GGAGTCTTGGGCCACAATACT |
| *Nape-Pld* | AGCGCCAAGCTATCAGTATCC | TCAGCCATCTGAGCACATTCG |
| *Ppar*α | AATGCCTTAGAACTGGATGACA | TGCAACTTCTCAATGTAGCCT |
| *Ppar*γ | ATGTCTCACAATGCCATCAGG | GTGATTTGTCCGTTGTCTTTCC |
| *Trpv1* | GTGGACAGCTACAGTGAGATAC | GCCACATACTCCTTGCGAT |
| *Gapdh* | AATGGTGAAGGTCGGTGTG | GTGGAGTCATACTGGAACATGTAG |

**Suppl. Table 2:**

List of lipid mediators analyzed

| **Name** | **Complete Name** | **Lipid Class** |
| --- | --- | --- |
| LEA | *N*-Linoleoyl-Ethanolamine | *N-*Acyl-Ethanolamine (NAE) |
| LEA-d4 | *N*-Linoleoyl-Ethanolamine-d4 | Internal Standard |
| DHEA | *N*-Docosahexaenoyl-Ethanolamine | *N*-Acyl-Ethanolamine (NAE) |
| DHEA-d4 | *N*-Docosahexaenoyl-Ethanolamine-d4 | Internal Standard |
| AEA-d4 | *N*-Arachidonoyl-Ethanolamine-d4 | Internal Standard |
| AEA-d8 | *N*-Arachidonoyl-Ethanolamine-d8 | Internal Standard |
| AEA | *N*-Arachidonoyl-Ethanolamine | *N*-Acyl-Ethanolamine (NAE) |
| PEA | *N*-Palmitoyl-Ethanolamine | *N*-Acyl-Ethanolamine (NAE) |
| PEA-d4 | *N-*Palmitoyl-Ethanolamine-d4 | Internal Standard |
| OEA | *N*-Oleoyl-Ethanolamine | *N*-Acyl-Ethanolamine (NAE) |
| OEA-d4 | *N*-Oleoyl-Ethanolamine-d4 | Internal Standard |
| SEA | *N*-Stearoyl-Ethanolamine | *N*-Acyl-Ethanolamine (NAE) |
| SEA-d3 | *N*-Stearoyl-Ethanolamine-d3 | Internal Standard |
| 1/2-SDG | 1/2-Stearidonoyl-Glycerol | Monoacyl-glycerol (MAG) |
| 1/2-EPG | 1/2-Eicosapentaenoyl-Glycerol | Monoacyl-glycerol (MAG) |
| 1-EPG-d5 | 1-Eicosapentaenoyl-Glycerol-d5 | Internal Standard |
| 1-DHG-d5 | 1-Docosahexaenoyl-Glycerol-d5 | Internal Standard |
| 1/2-DHG | 1/2-Docosahexaenoyl-Glycerol | Monoacyl-glycerol (MAG) |
| 1/2-LG | 1/2-Linoleoyl-Glycerol | Monoacyl-glycerol (MAG) |
| 1-LG-d5 | 1-Linoleoyl-Glycerol-d5 | Internal Standard |
| 1-AG-d5 | 1-Arachidonoyl-Glycerol-d5 | Internal Standard |
| 1/2-AG | 1/2-Arachidonoyl-Glycerol | Monoacyl-glycerol (MAG) |
| 1-DPG-d5 | 1-Docosapentaenoyl-Glycerol-d5 | Internal Standard |
| 1/2-DPG | 1/2-Docosapentaenoyl-Glycerol | Monoacyl-glycerol (MAG) |
| 1/2-OG | 1/2-Oleoyl-Glycerol | Monoacyl-glycerol (MAG) |

**Suppl. Table 3** Quantification range of tryptophan and tryptophan metabolites in the plasma sample analysis. LLOQ: lower limit of quantification; ULOQ: upper limit of quantification.

|  | **LLOQ (nM)** | **ULOQ (nM)** |
| --- | --- | --- |
| Picolinic acid | 7.5 | 750 |
| Qunolinic acid | 4.5 | 3000 |
| 3-OH-kynurenine | 5 | 1000 |
| Serotonin | 22.5 | 15,000 |
| 5-OH-tryptophan | 0.5 | 100 |
| Kynurenine | 15 | 10,000 |
| Tryptamine | 1 | 100 |
| 3-OH-anthranilic acid | 5 | 1000 |
| Tryptophan | 300 | 200,000 |
| 5-OH-indole acetic acid | 3 | 2000 |
| 3-indoxylsulfate | 112.5 | 75,000 |
| N-acetyl-serotonin | 0.5 | 100 |
| Xanthurenic acid | 0.75 | 500 |
| Indole-3-acetamide | 1 | 100 |
| Kynurenic acid | 2.5 | 500 |
| Indole-3-lactic acid | 50 | 10,000 |
| Indole-3-aldehyde | 5 | 500 |
| Indole-3-acetic acid | 22.5 | 15,000 |
| Tryptophol | 1.25 | 250 |
| Melatonin | 0.5 | 100 |
| Indole-3-propionic acid | 22.5 | 15,000 |

**Suppl. Table 4.** Quantification range of tryptophan and tryptophan metabolites in the brain and liver sample analysis. LLOQ: lower limit of quantification; ULOQ: upper limit of quantification.

|  | **LLOQ (nM)** | **ULOQ (nM)** |
| --- | --- | --- |
| Picolinic acid | 7.5 | 5000 |
| Qunolinic acid | 15 | 3000 |
| 3-OH-kynurenine | 5 | 1000 |
| Serotonin | 3 | 2000 |
| 5-OH-tryptophan | 3.75 | 750 |
| Kynurenine | 1.5 | 1000 |
| Tryptamine | 0.75 | 500 |
| 3-OH-anthranilic acid | 1.5 | 1000 |
| Tryptophan | 75 | 50,000 |
| 5-OH-indole acetic acid | 3 | 2000 |
| 3-indoxylsulfate | 37.5 | 25,000 |
| N-acetyl-serotonin | 0.15 | 100 |
| Xanthurenic acid | 0.375 | 250 |
| Indole-3-acetamide | 0.15 | 100 |
| Kynurenic acid | 0.375 | 250 |
| Indole-3-lactic acid | 3.75 | 2500 |
| Indole-3-aldehyde | 1.5 | 1000 |
| Indole-3-acetic acid | 1.5 | 1000 |
| Tryptophol | 0.75 | 500 |
| Melatonin | 0.15 | 100 |
| Indole-3-propionic acid | 1.5 | 1000 |

**Suppl. Figure 1 Animal experimental design**


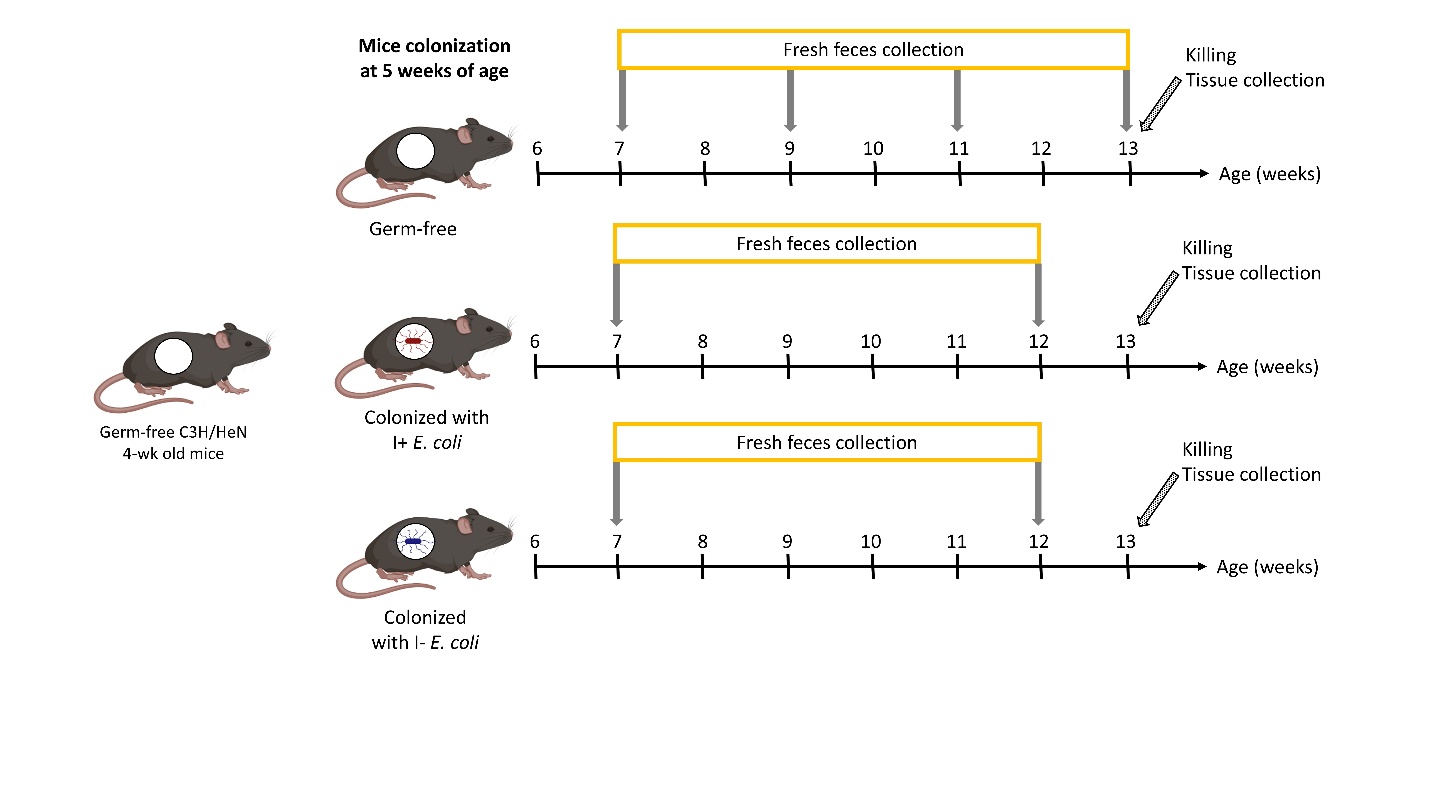


The timeframe for the various interventions is indicated. Fecal samples were taken at the time points indicated by the gray arrows to verify germ-free status (germ-free mice), or to enumerate the bacterial population and determine tryptophan and indole concentrations (mice colonized with E. coli).

**Suppl. Figure 2A**

**
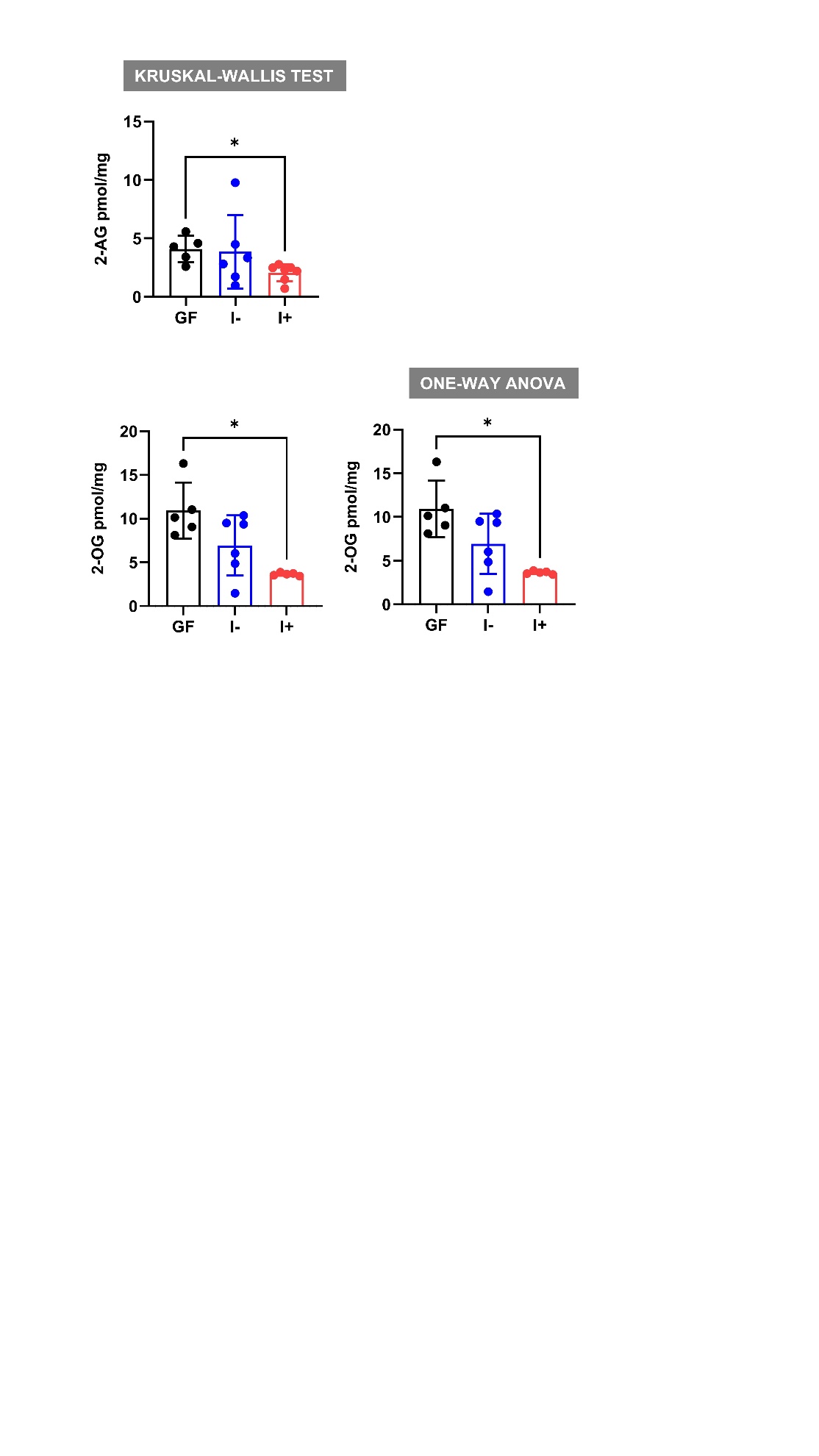
**

**Suppl. Figure 2B**

**
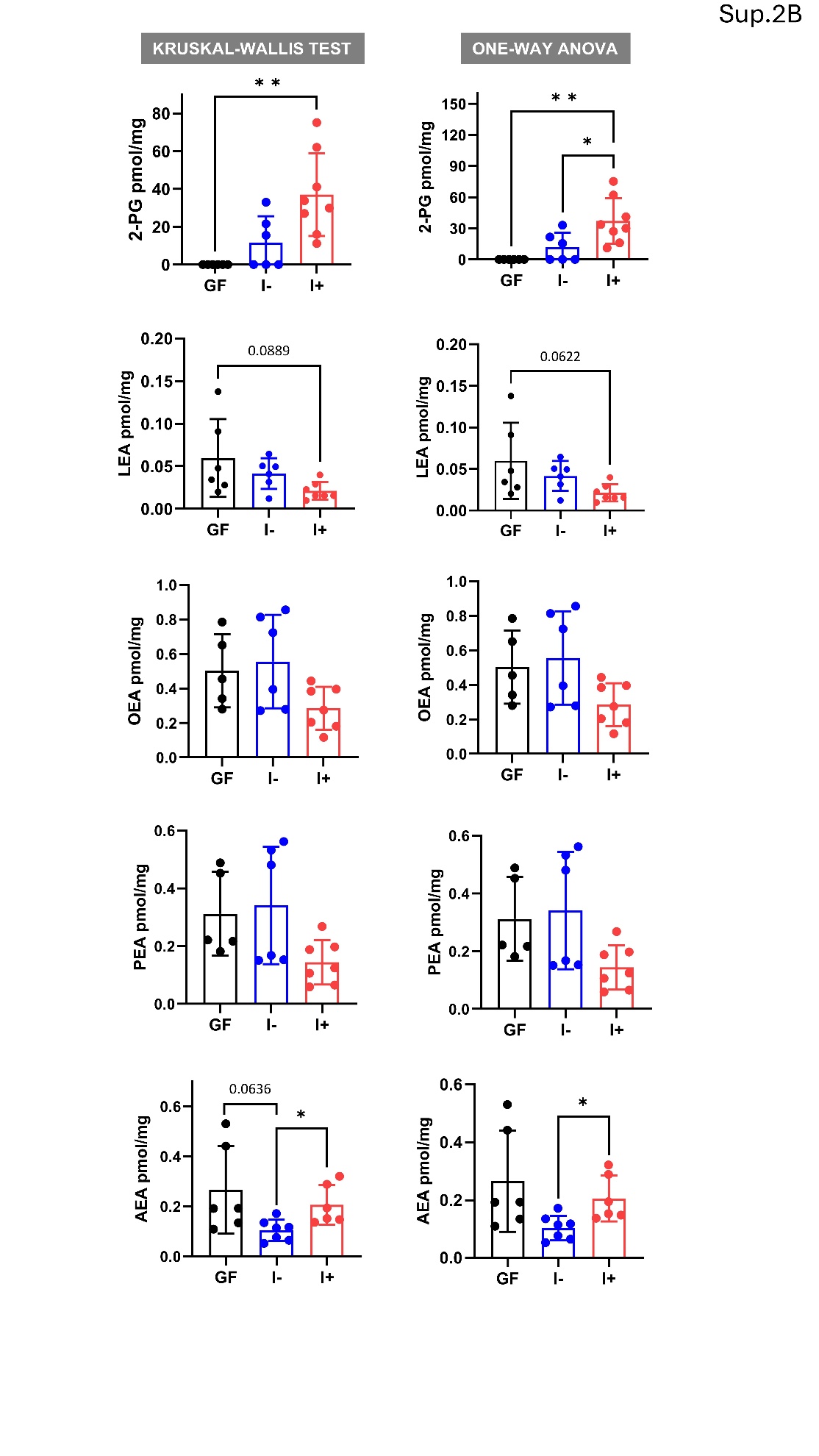
**

**Suppl. Figure 2C**

**
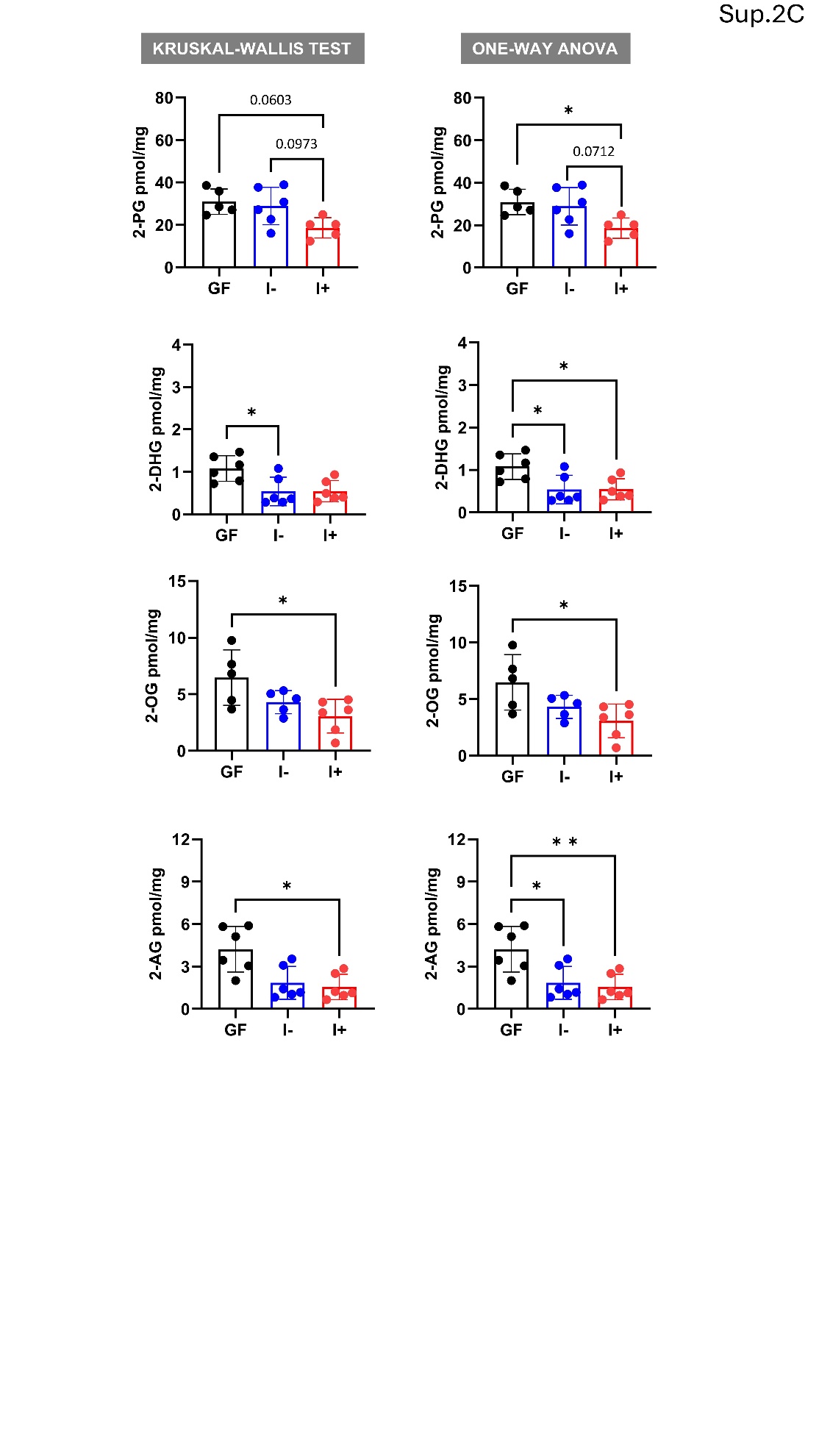
**

**Suppl. Figure 3A**

**
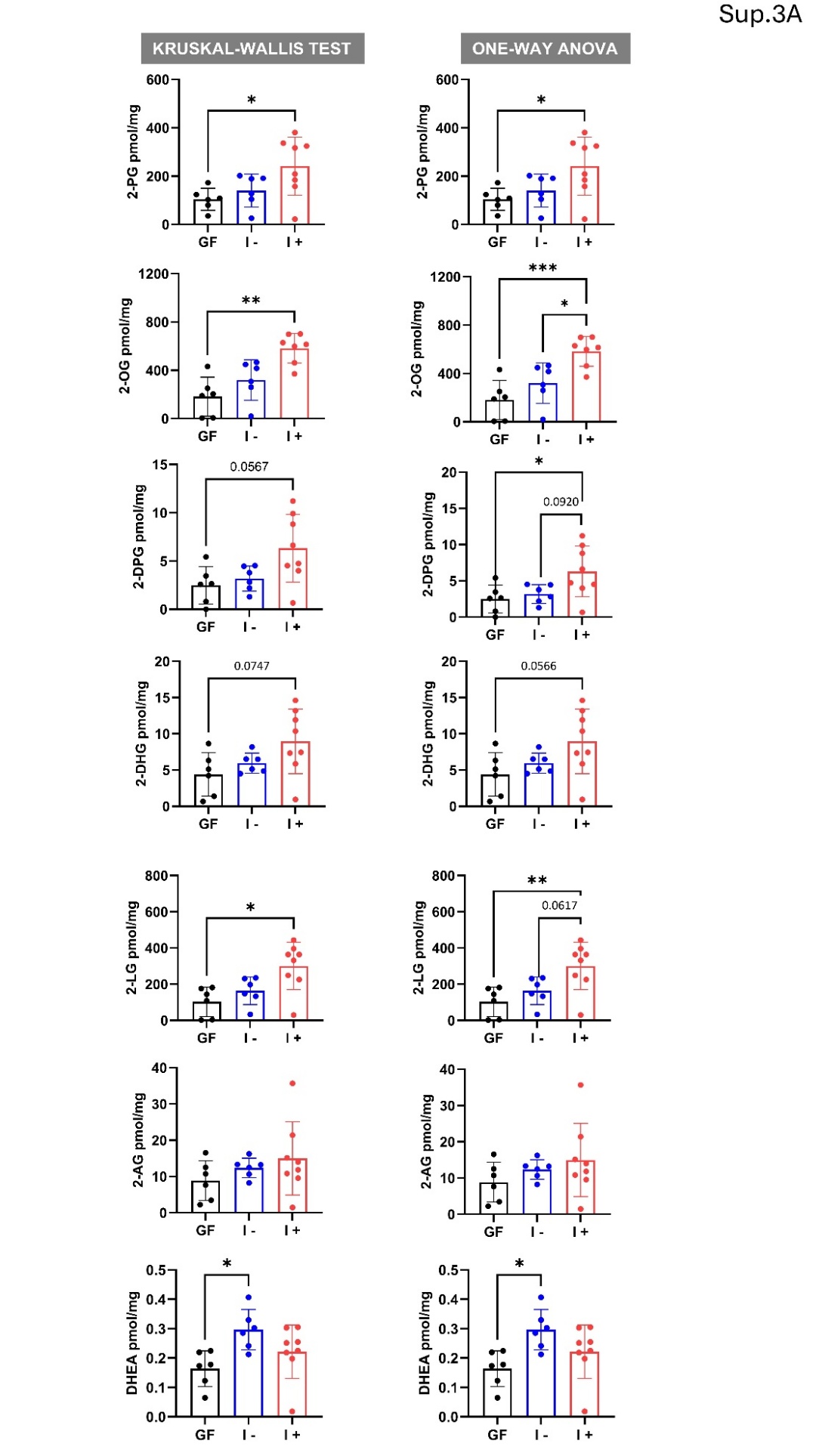
**

**Suppl. Figure 3B**

**
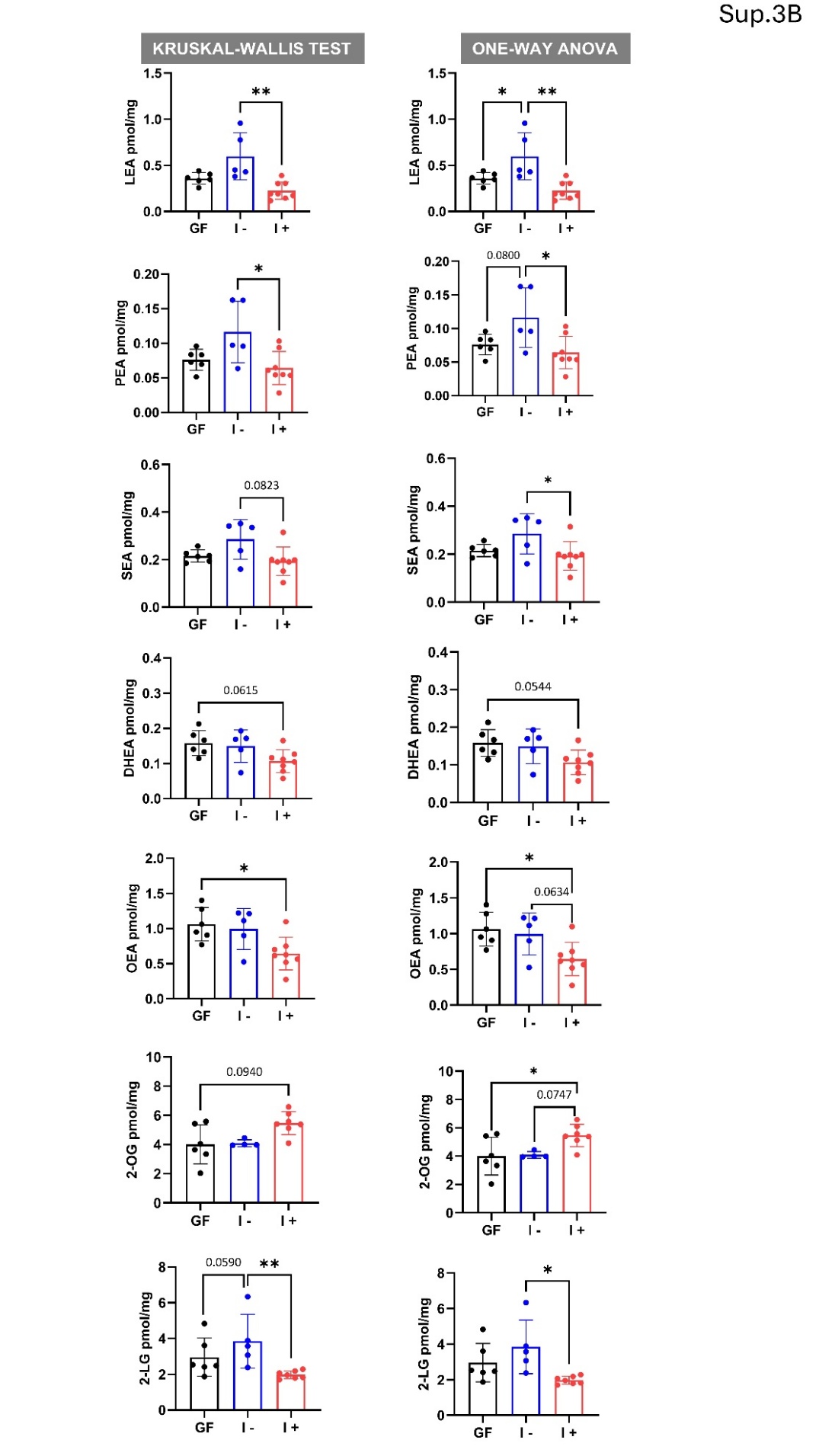
**

**Supl. Figure 3C**

**
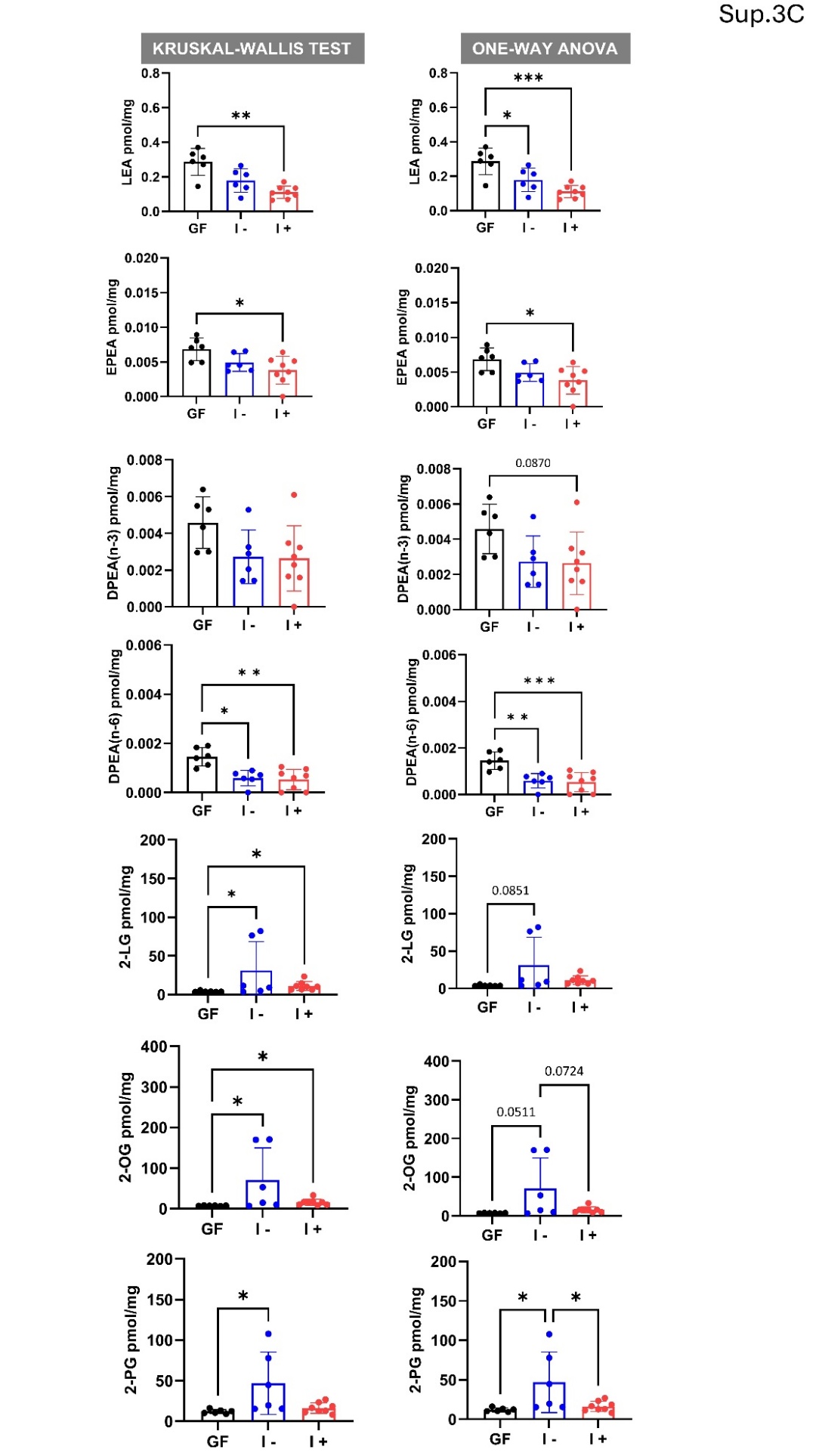
**

**Suppl. Figure 3D**

**
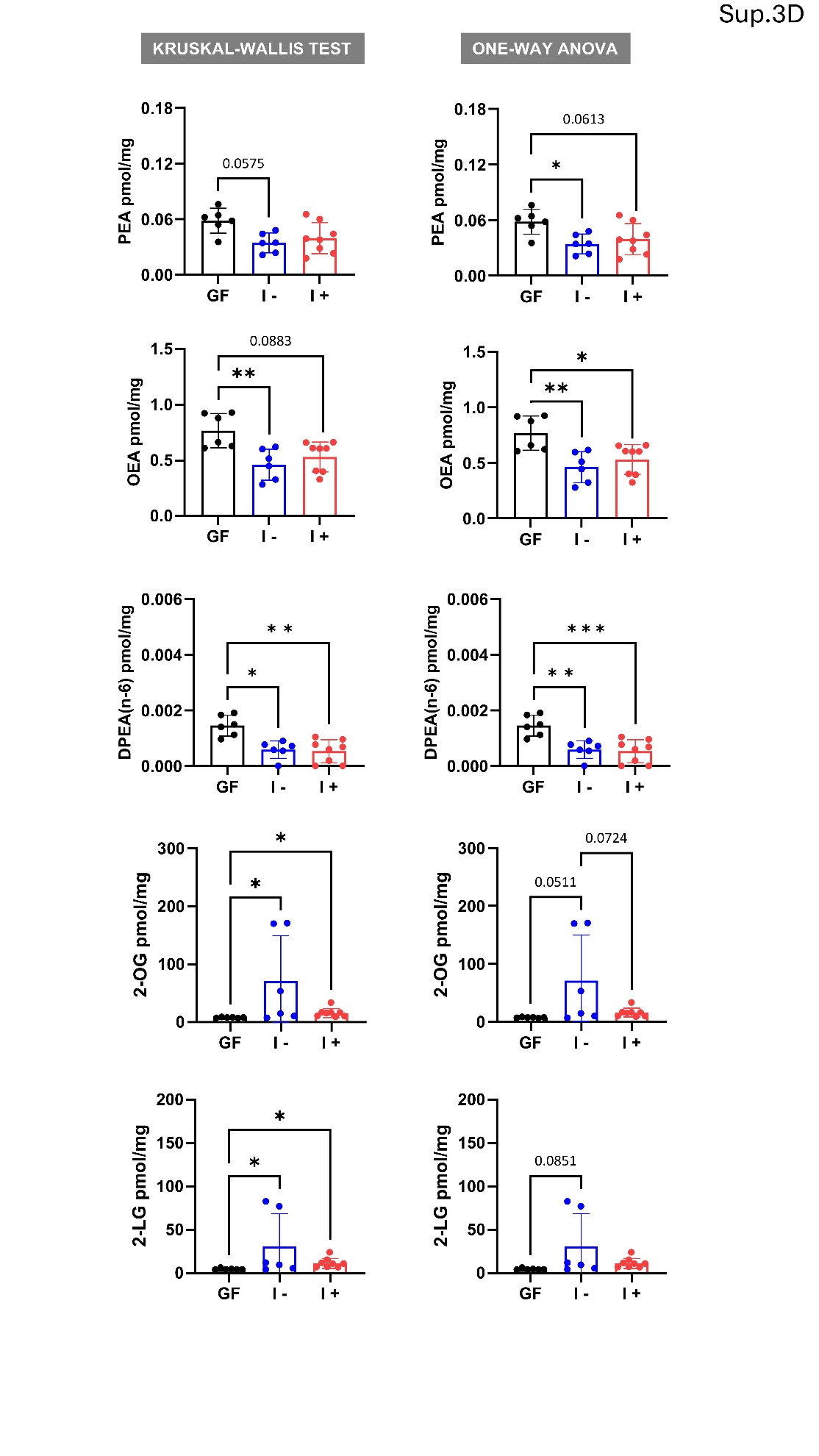
**

**Suppl. Figure 4A**

**
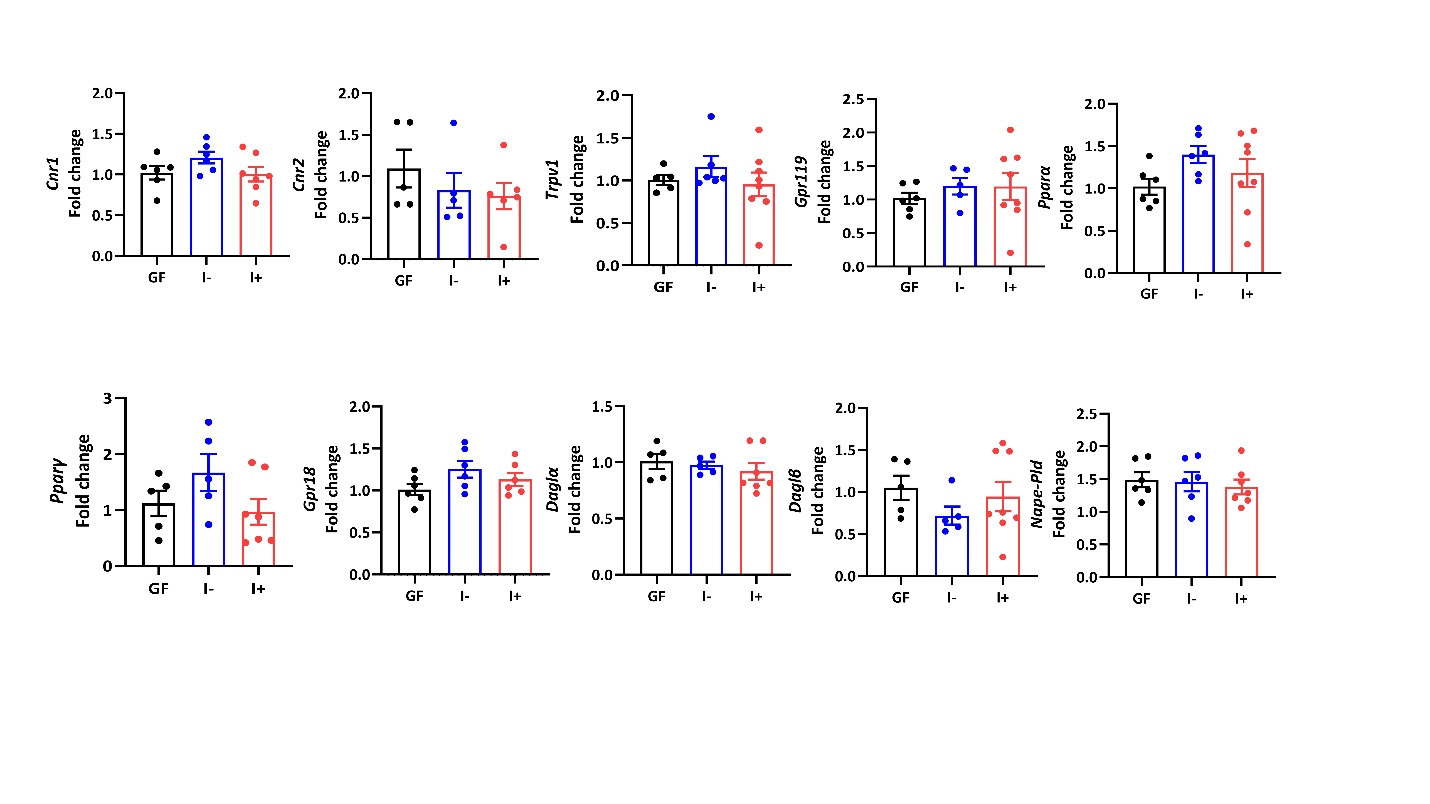
**

**Suppl. Figure 4B**

**
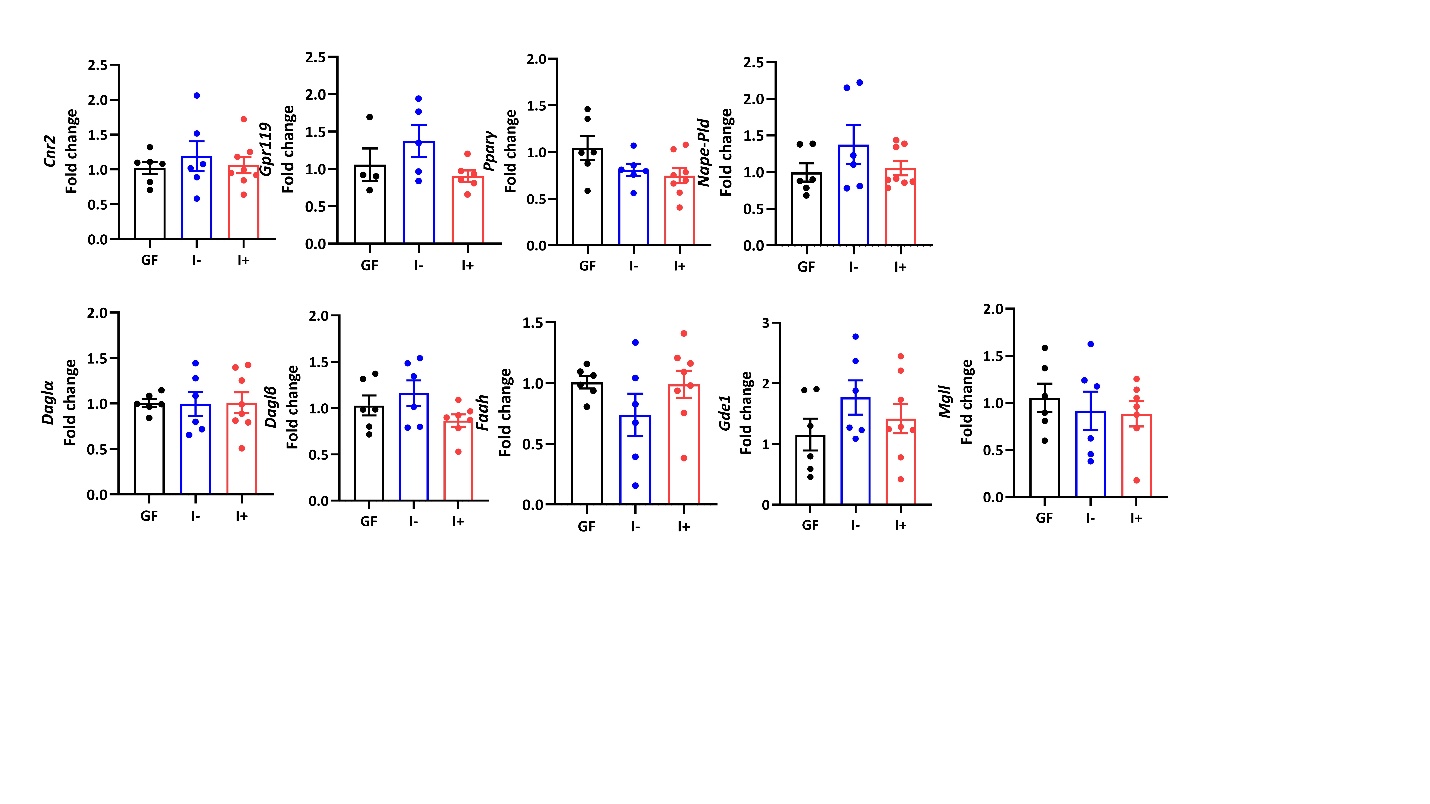
**

**Suppl. Figure 5A**

**
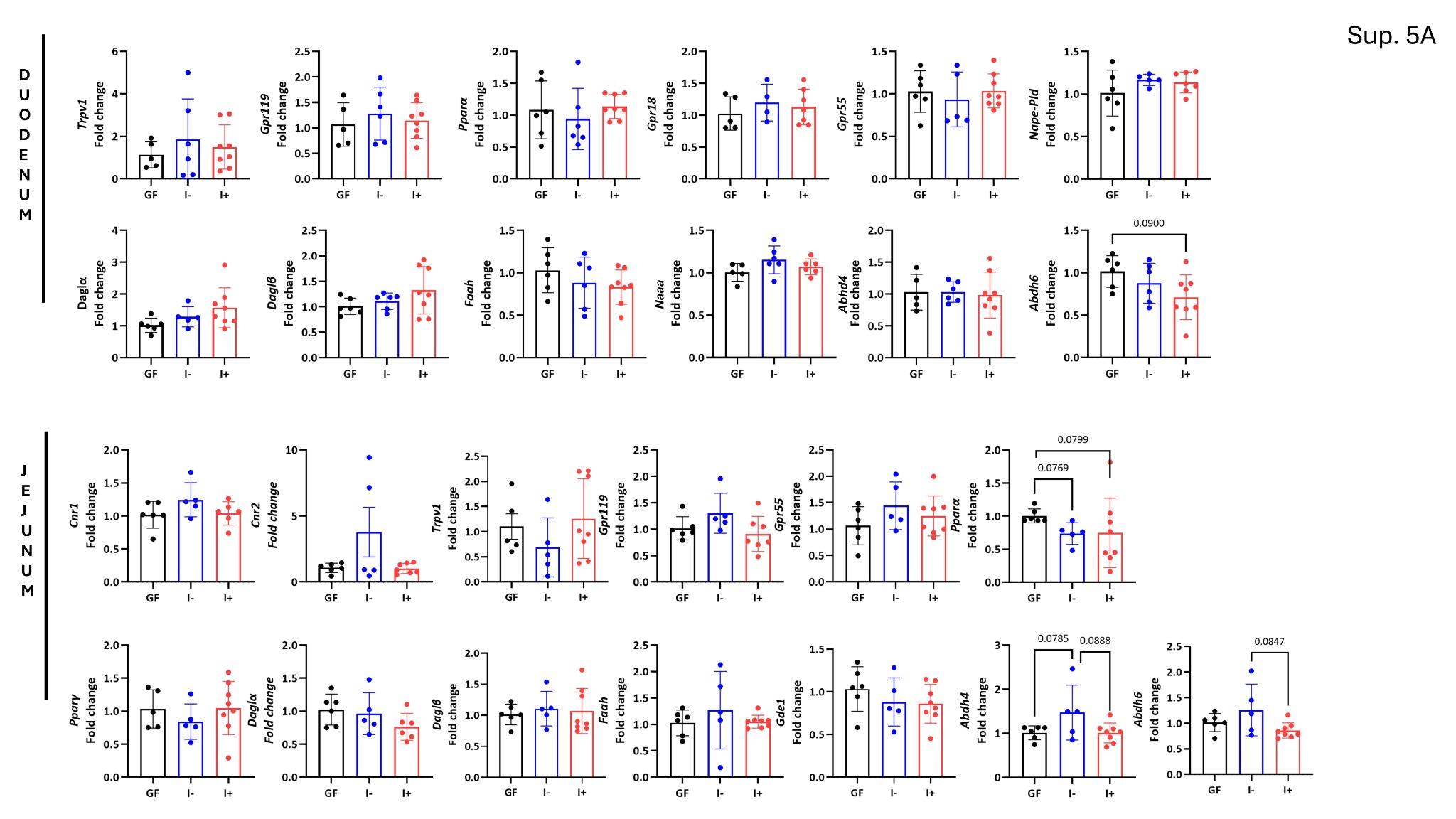
**

**Suppl. Figure 5B**

**
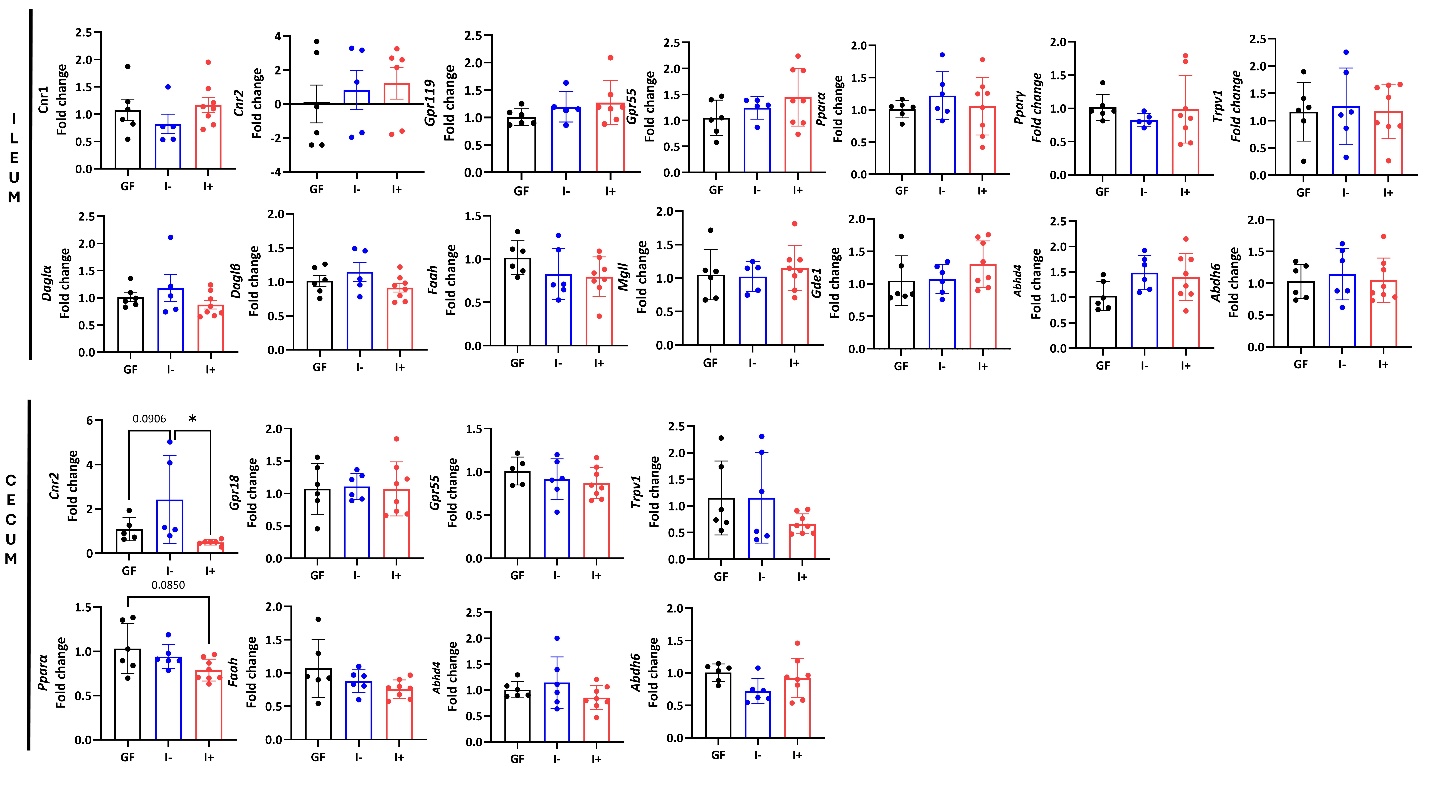
**

**Suppl. Figure 5C**

**
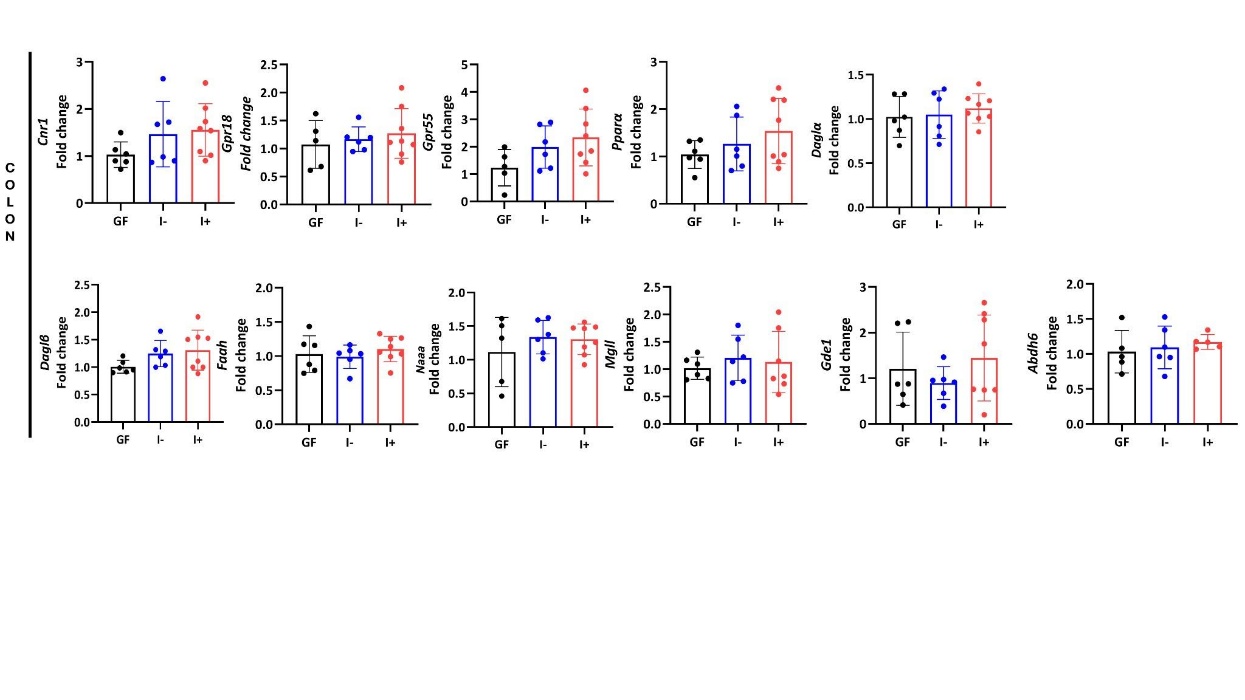
**

**Suppl. Figure 6**

**
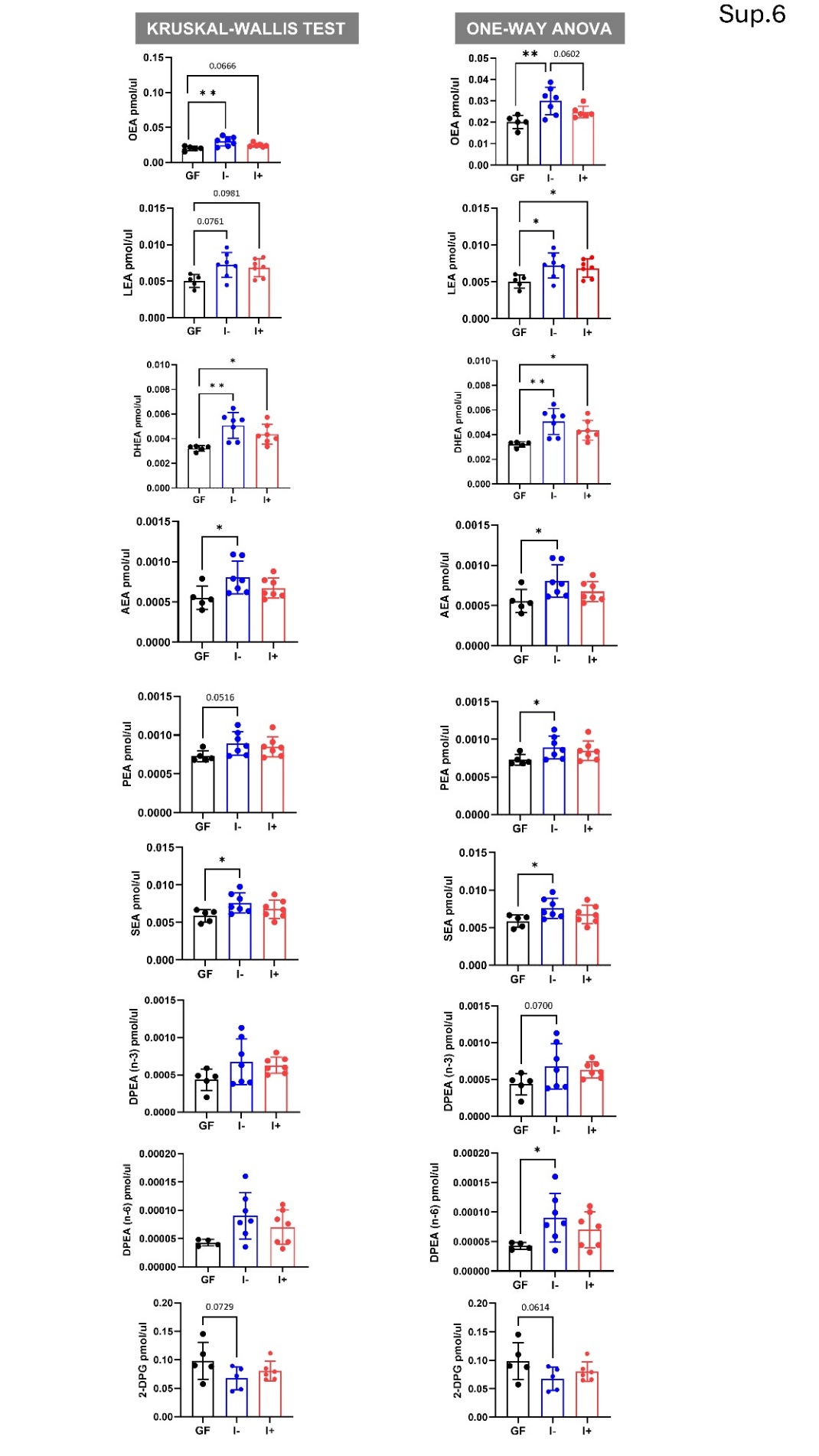
**
